# A high-quality chromosome-level *Eutrema salsugineum* genome, an extremophile plant model

**DOI:** 10.1186/s12864-023-09256-x

**Published:** 2023-04-05

**Authors:** Meng Xiao, Guoqian Hao, Xinyi Guo, Landi Feng, Hao Lin, Wenjie Yang, Yanyu Chen, Kexin Zhao, Ling Xiang, Xinyao Jiang, Dong Mei, Quanjun Hu

**Affiliations:** 1 Key Laboratory for Bio-Resource and Eco-Environment of Ministry of Education & Sichuan Zoige Alpine Wetland Ecosystem National Observation and Research Station, College of Life Science, Sichuan University, Chengdu, China; 2 Faculty of Agriculture, Forestry and Food Engineering, Yibin University, Yibin, 644007 Sichuan China

**Keywords:** *Eutrema salsugineum*, Adaptation, De novo genome, Halophyte, Genome assembly

## Abstract

**Background:**

*Eutrema salsugineum* (2n = 14), a halophyte in the family Brassicaceae, is an attractive model to study abiotic stress tolerance in plants. Two versions of E. salsugineum genomes that previously reported were based on relatively short reads; thus, the repetitive regions were difficult to characterize.

**Results:**

We report the sequencing and assembly of the *E. salsugineum* (Shandong accession) genome using long-read sequencing and chromosome conformation capture data. We generated Oxford Nanopore long reads at high depth (> 60X) of genome coverage with additional short reads for error correction. The new assembly has a total size of 295.5 Mb with 52.8% repetitive sequences, and the karyotype of *E. salsugineum* is consistent with the ancestral translocation Proto-Calepineae Karyotype structure in both order and orientation. Compared with previous assemblies, this assembly has higher contiguity, especially in the centromere region. Based on this new assembly, we predicted 25,399 protein-coding genes and identified the positively selected genes associated with salt and drought stress responses.

**Conclusion:**

The new genome assembly will provide a valuable resource for future genomic studies and facilitate comparative genomic analysis with other plants.

**Supplementary Information:**

The online version contains supplementary material available at 10.1186/s12864-023-09256-x.

## Introduction

Soil salinization has become one of the major environmental and socioeconomic issues globally, and climate change influences the dynamics of naturally occurring soil salinization [1]. Cultivating marginal lands or long-term irrigation, which leads to the accumulation of salt, will expose crops to adverse conditions, thus reducing the yield of agriculturally and economically important plants [2]. New varieties of current crops or new crops are therefore needed to sustain agriculture in many regions of the world. In addition to regulating the expression of specific genes in plants, the transfer of one or more genes between species is also promising [3–5]. Identification of genetic elements underlying adaptation to salinity in species that exhibit natural tolerance can provide crucial insights into mechanisms that confer high levels of salt tolerance.

*Eutrema salsugineum*, previously named *Thellungiella salsuginea* [6], is an attractive model to study the mechanisms of abiotic stress tolerance in plants [7, 8]. As an extremophile plant, *E. salsugineum* can survive after exposure to extreme salinity (500 mm NaCl) or cold to − 15 °C and reproduce normally [9]. Being closely related to the model plant *Arabidopsis thaliana, E. salsugineum* shares many beneficial attributes, such as small size, self-fertility, short life cycle, small genome size and genetic transformation [10, 11]. Previous studies have generated two versions of genome assemblies for the best-studied Shandong ecotype using Illumina and Sanger sequencing respectively [12, 13]. However, short-read sequencing technologies often yield incomplete and highly fragmented genome assemblies, especially for plant genomes that are featured by abundant genomic repeats and whole-genome duplications [14]. Although both assemblies assigned the assembled scaffold to seven pseudomolecules based on the chromosomal comparative painting (CCP)-derived karyotype [15], they are quite fragmented, especially in the centromere region. Thus, the de novo assembly of a new reference genome for *E. salsugineum* using long reads is imperative. Recently, commercialized long-read technologies (Pacific Biosciences and Oxford Nanopore) can sequence long DNA fragments (> 15 kilobases on average) and hold great promise for producing high-quality genomes in terms of contiguity and completeness of repetitive regions [16, 17]. Combining these methods with additional scaffolding, such as optical mapping and chromosome conformation capture (Hi-C), has been successful in achieving chromosome-level assemblies for several plant genomes [18–23].

In the present study, we report an improved, highly contiguous reference genome assembly of *E. salsugineum* ecotype Shandong (hereafter EsaV3) by combining Oxford Nanopore long-read sequencing and Hi-C technology. We compared this new assembly with previous versions, analyzed expanded/contracted gene families and identified positively selected genes.

## Results

### Genome sequencing, de novo assembly, and annotation

We estimated the genome size to be 258 Mb by k-mer analysis (Supplementary Fig. 1). To assemble the *E. salsugineum* (Shandong ecotype) genome, we employed a strategy that combined Oxford Nanopore long-read sequencing and Hi-C scaffolding, with high-coverage Illumina reads for error correction.

**Fig. 1:**
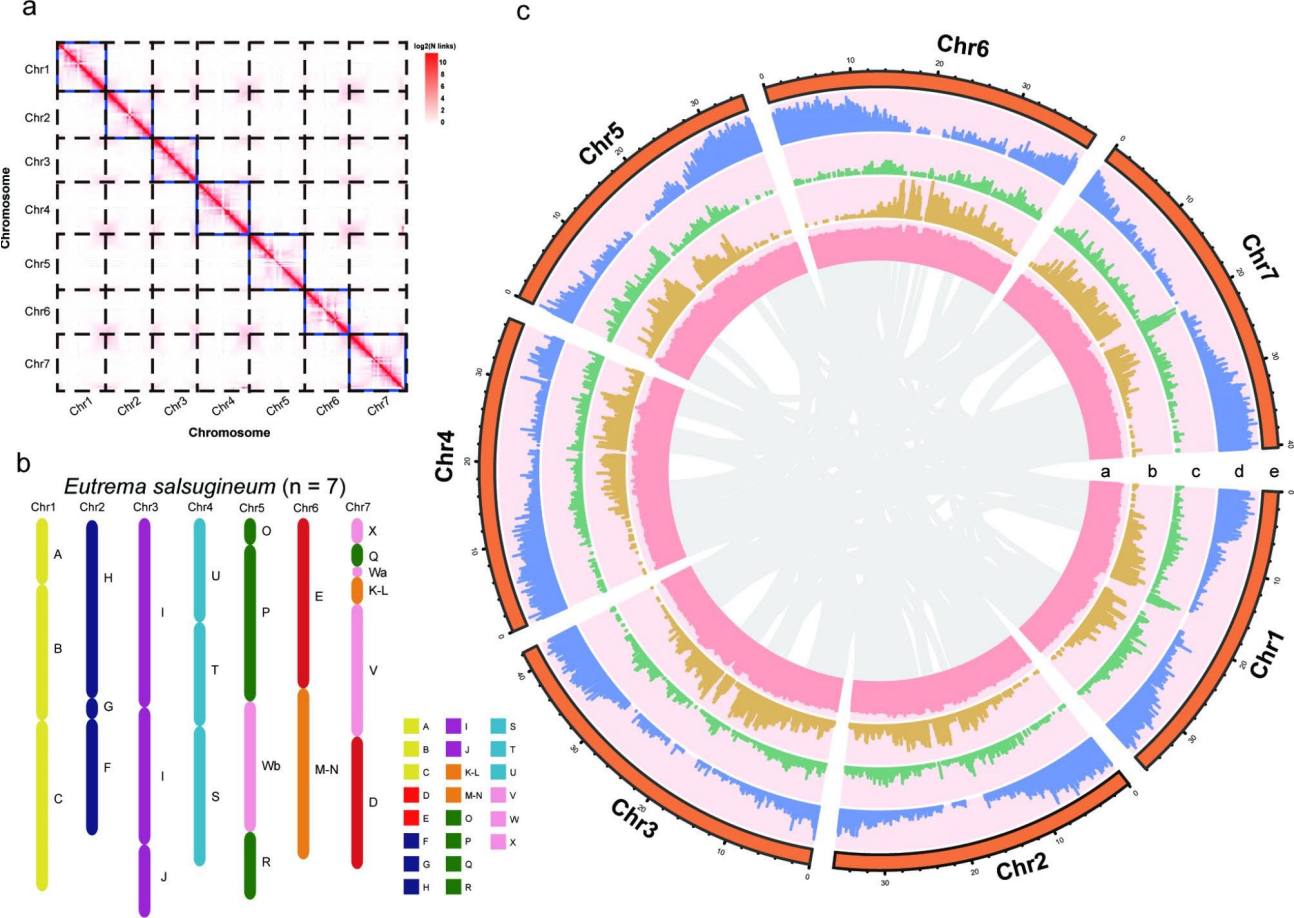
**Hi-C assisted assembly of EsaV3 pseudochromosomes**. **a**. Hi-C chromatin interaction heatmap for the 7 pseudochromosomes of the *E. salsugineum* genome at a resolution of 200 kb. **(b)** Genome structure of *E. salsugineum*. The 22 ancestral genomic blocks are indicated by capital letters (A-X) and are colored based on their position in the eight chromosomes of the Ancestral Crucifer Karyotype (ACK). **(c)** The landscape of the genome assembly and annotation of EsaV3. Tracks from inside to the outside correspond to a, GC content; b, Copia density; c, Gypsy density; d, gene density; and e, seven pseudochromosomes

We generated a total of 19.40 Gb of long-read sequencing data for *E. salsugineum* (Shandong ecotype) with four flow cells by Oxford Nanopore Technologies (ONT). After quality control, 17.57 Gb clean reads (~ 63× genome coverage) were retained and had an average length of 19.69 kb, an N50 of 28.36 kb and the longest read being 176.7 kb (Supplementary Table 1). Our initial assembly of the long read data by Canu [24] software resulted in 297.1 Mb of sequences within 1,244 contigs that were longer than 1 kb (Supplementary Table 2). Of these, 16 contigs showed extraordinarily high GC content (> 0.6) and were thus removed from further analysis (Supplementary Fig. 2). To correct assembly errors induced by long-read sequencing data, we also generated Illumina short reads (18.06 Gb, ~ 65× coverage) for the same individual. After two rounds of Pilon [25] correction, the final contig assembly had an N50 of 3.1 Mb and the longest sequence being more than 17 Mb (Supplementary Table 2). Finally, we mapped the Hi-C data (39.51 Gb, 141× coverage) onto the assembled contigs with the modified 3D-DNA [26] and Juicebox Assembly Tools (JBAT) [27] workflow, which split the input contigs into 1,479 (sub)contigs and clustered 617 of them into seven chromosome-scale superscaffolds with a total length of 265.7 Mb (Fig. 1a,b).

Additionally, we evaluated the quality of the genome assembly using high-quality short reads and near-universal single-copy orthologs. We found that 96.1% of the short reads could be reliably aligned to the genome assembly, with 88.6% being properly aligned to the genome with their mates. Benchmarking Universal Single-Copy Orthologs (BUSCO) [28] analysis showed that 99.1% of conserved BUSCO proteins were detected in the *E. salsugineum* assembly, including 0.2% of fragment BUSCO proteins (Supplementary Table 4). We also used Merqury [29] software to evaluate the consensus quality (QV) value and the completeness of the assembled genome. Higher QV indicates a more accurate consistency, where Q30 indicates 99.99% accuracy and Q40 indicates 99.99% accuracy. The assembly of *E. salsugineum* has a QV score of 31.35 and completeness of 97.3%, indicating the assembled genome has a high base accuracy.

**Table 1:** A comparison of the three *E. salsugineum* genome assemblies

Assembly feature	EsaV1 (NCBI_TsV2-8)	EsaV2 (Phytozome_173_v1)	EsaV3 (This study)
Assembly length	231.9 Mb	243.1 Mb	295.5 Mb
Total ungapped length	208.9 Mb	238.4 Mb	295.1 Mb
Percentage of gaps	9.92%	1.93%	0.14%
BUSCO score for assembly	97.4%	99.2%	99.1%
Number of scaffolds	2663	638	655
Scaffold N50	0.40 Mb	13.44 Mb	36.82 Mb
Scaffold L50	117	8	4
Number of contigs	28,682	3,658	1,479
Contig N50	0.03 Mb	0.22 Mb	3.05 Mb
Contig L50	1,496	311	29
Predicted gene models	28,457	26,351	25,399
BUSCO score for predicted gene models	95.8%	99.1%	97.2%
Total exon	149,079	137,652	136,411
Exons per gene	5.23	5.22	5.37
Mean gene length	2,041 bp	2,209 bp	2,559 bp

### Comparison of three versions of *E. salsugineum* genomes

Compared with previous assemblies based on Illumina (EsaV1) and Sanger (EsaV2) methods, our novel EsaV3 captured more sequences and showed less fragmentation, as indicated by the number and N50 length of contigs, improving sequence contiguity by 102- and 14-fold, respectively. Our assembly EsaV3 is larger than both previous genome versions (231.89 Mb and 243 Mb, respectively; Table 1). The contig N50 value of the new assembly, obtained by combining Nanopore long reads and Hi-C data, is 3.05 Mb, which is ~ 102-fold and ~ 14-fold greater than the corresponding values for EsaV1 and EsaV2 (0.03 kb and 0.22 kb, respectively; Table 1). Moreover, the scaffold N50 value of our EsaV3 is 36.82 Mb, which is better than those of previous assemblies (0.40 Mb and 13.44 Mb, respectively; Table 1). Compared with EsaV1 and EsaV2, the integrity and continuity of the novel assembled genome are significantly improved, and the annotated gene structure is more complete.

**Fig. 2:**
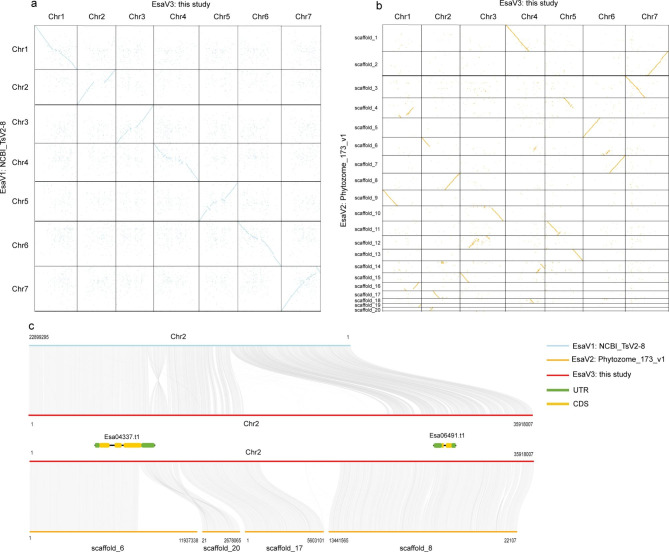
**Synteny comparative analysis in three*E. salsugineum*genome assemblies. a** Genome-wide dot plot for EsaV1 and EsaV3. **b** Genome-wide dot plot for EsaV2 and EsaV3. **c** An example of a region comparison in three *E. salsugineum* genome assemblies. Two of the newly annotated genes in our EsaV3 assembly compared with EsaV1 and EsaV2.

We performed whole-genome alignment of three genome assemblies of *E. salsugineum* using LAST [30] alignment software. The dot plot reveals that the EsaV1 genome has a one-to-one correspondence with the pseudochromosomes of our EsaV3 assembly, thus confirming the results from comparative chromosome painting (Fig. 2a). Furthermore, the centromere regions are more complete in EsaV3 than in EsaV1, which explains why the assembled genome size of EsaV3 is larger than that of EsaV1. Although the genome of EsaV2 is fragmented, we recovered corresponding relationships between scaffolds and chromosomes in EsaV3 (Fig. 2b,c).

We performed whole-genome synteny alignment of gene pairs for the protein sequences of the current reference genome of *E. salsugineum* (EsaV2) and our assembly (Supplementary Fig. 4). Although the genes annotated in EsaV2 have good correspondence in our EsaV3 assembly, gaps near the centromere region exist in the EsaV2 assembly compared with EsaV3. In addition, we found that the gene structure annotated in our EsaV3 was more complete than that in EsaV2 (Supplementary Figs. 5 and 6).

### LTR insertion

Repetitive regions spanning ~ 156.1 Mb (52.83% of the assembly size) in our EsaV3 genome were identified using a combination of homology-based and de novo approaches (Supplementary Table 5). Long terminal repeat (LTR) retrotransposons are the most abundant repetitive elements in our novel assembly, which is mainly composed of Gypsy-LTRs and Copia-LTRs, accounting for 14.20% and 5.82% of the genome, respectively (Supplementary Table 6). We also used the Circos [31] tool (http://www.circos.ca) to visualize the GC content, Copia density, Gypsy density and gene density of each chromosome. The density of Copia was high in the centromeric region of the genome (Fig. 1c).

**Fig. 3:**
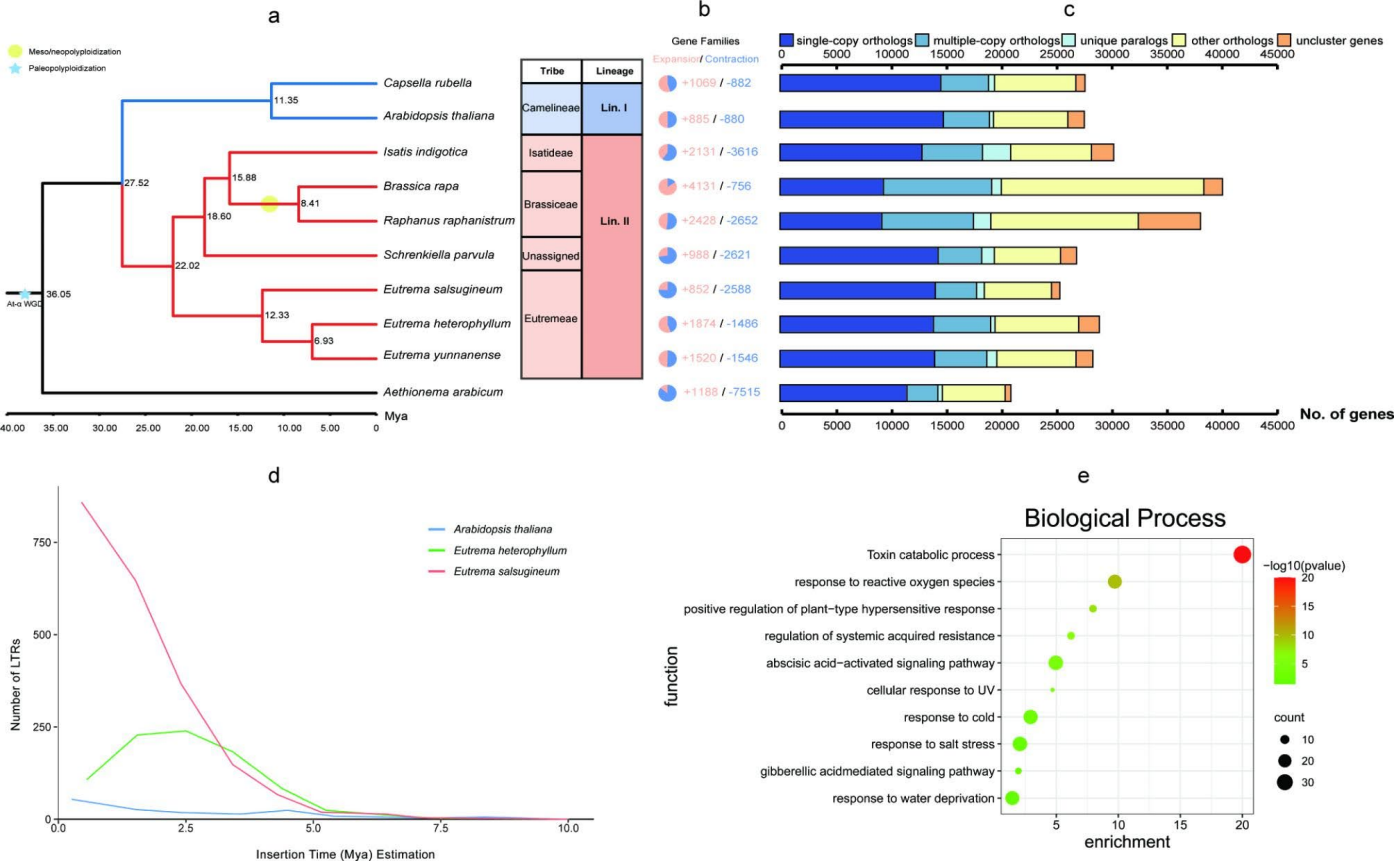
**Evolutionary and comparative genomic analyses of the newly assembled*E. salsugineum*genome. a** Phylogenetic relationships and divergence times of *E. salsugineum* and nine other Brassicaceae species with *A. arabicum* as the outgroup. The blue pentagram represents the At-α WGD event shared by all Brassicaceae species, and the yellow circle represents a Brassicae-specific triplication. The estimated divergence times (million years ago, Mya) are indicated at each node of the phylogenetic tree. **b** Expansions and contractions of gene families. The colors in light pink and dark blue indicate the expanded and contracted gene families, respectively. **c** Clusters of orthologous and paralogous gene families in *E. salsugineum* and nine more fully sequenced plant genomes. Gene families were identified using the OrthoFinder package with the default parameters. **d** Estimated insertion times of LTR retrotransposons within *A. thaliana*, *E. heterophyllum*, and *E. salsugineum*. **e** GO enrichment analysis bubble plot of significantly expanded genes in *E. salsugineum*

To further analyze the evolutionary dynamic history of LTR retrotransposons in the genome, we estimated the insertion time of the intact LTR-RTs in three closely related species of the family Brassicaceae (*A. thaliana* [32], *Eutrema heterophyllum* [33] and *E. salsugineum*) ((Fig. 3d and Supplementary Table 6). We also estimated the time of insertion for LTR/Gypsy, LTR/Copia, and unknown LTR-RTs (Supplemental Fig. 9). As both *E. heterophyllum* and *E. salsugineum* experienced a recent burst of LTR retrotransposon amplification, we constructed the phylogenetic trees of LTR/Copia and LTR/Gypsy based on the RT domains of LTR-RTs between *E. heterophyllum* and *E. salsugineum* to compare the species-specific LTR-RTs by Tesorter [34] (Fig. 3d and Supplementary Fig. 8). The results showed that most clades of LTR/Gypsy and LTR/Copia have more members in *E. salsugineum* than in *E. heterophyllum* (Supplemental Table 7). Furthermore, the Galaderiel family of LTR/Gypsy and the Angela family of LTR/Copia are only found in *E. salsugineum*. Comparison of specific and non-specific LTR-RTs between *E. salsugineum* and *E. heterophyllum* revealed that both specific and non-specific LTR-RTs have expanded in the *E. salsugineum* genome, especially species-specific LTR-RTs (Supplemental Table 8). These results suggest that the dramatic LTR insertion is responsible for the relatively large genome of *E. salsugineum* (295 Mb) [35].

Previous studies have also shown that transposons may play a role in adaptive evolution [36]. We searched for genes near the location of the recent LTR insertion in *E. salsugineum* and performed GO enrichment analysis of these genes. The results show that they are significantly enriched in DNA integration and enzyme activities (Supplementary Tables 11 and 12; Supplementary Fig. 10).

### Gene prediction and annotation

Using a combination of homologous protein alignment, de novo prediction, and transcript mapping, we predicted 25,399 protein-encoding genes in the EsaV3 genome, with an average sequence length of 2,559 bp. The average gene length of EsaV3 (2,559 bp) is greater than that of EsaV1 (2,041 bp) and EsaV2 (2,209 bp). The protein-encoding genes have 5.37 exons, and each exon is 295 bp long on average (Supplementary Table 10). The gene length distribution was similar between the three genome assemblies (Supplementary Fig. 7). Moreover, through cluster analysis of three versions of *E. salsugineum* genomes, a total of 1,153 newly annotated genes, including *ARF1* [37], *RPP2D* [38], *ARA12* [39] and *RLP12* [40], were identified in our *E. salsugineum* genome, which was mainly related to transmembrane transport or salt stress. The newly annotated genes have a high density in the distal end of the chromosomes (Supplementary Fig. 11). The GO enrichment results show that these genes are mainly related to toxin catabolic processes and DNA integration (Supplementary Tables 11 and Supplementary Fig. 13).

We annotated genes based on homology for 74.11% and 96.04% of the genes in the Swiss-Prot [41] and TrEMBL [42] databases, respectively. In addition, approximately 78.89%, 70.72%, and 40.10% of the protein-encoding genes were successfully annotated by using the InterPro [43], Gene Ontology (GO) [44] and Kyoto Encyclopedia of Genes and Genomes (KEGG) [45] pathway databases, respectively. Overall, 24,448 of the 25,399 protein-coding genes (96.26%) were assigned functional annotations in the EsaV3 genome (Supplementary Table 14).

### Phylogenetic relationships and WGD analyses

We clustered the annotated *E. salsugineum* genes into gene families with those of *A. thaliana* [32], *Aethionema arabicum* [46], *Capsella rubella* [47], *Brassica rapa* [48], *Schrenkiella parvula* [49], *Isatis indigotica* [50], *Raphanus raphanistrum* [51], *E. heterophyllum* [33], and *Eutrema yunnanense* [33] by OrthoFinder [52] with *A. arabicum* [46] as the outgroup. The most recent common ancestor (MRCA) of the 10 species contained 25,500 gene families and 2,355 single-copy orthologous genes (Fig. 3c). These single-copy orthologous genes among ten species were selected to build a phylogenetic tree using the maximum likelihood method. We used MCMCTree [53] with fossil calibration to estimate species divergence times. Phylogenetic analysis indicates that *E. salsugineum* is most closely related to *E. heterophyllum-E. yunnanense* branch and belongs to tribe Eutremeae in Lineage II [54, 55]of Brassicaceae. The estimated divergence time between *E. salsugineum* and *E. heterophyllum*-*E. yunnanense* branch is estimated to have occurred 12.33 million years ago (Mya) (Fig. 3a).

The occurrence of whole-genome duplication (WGD) or polyploidization provides the original genetic material for biological evolution and promotes biological evolution to a large extent [56]. We used synonymous substitution rates (Ks) between paralogous gene pairs to identify potential WGD events. The distribution of Ks between syntenic blocks suggested that *E. salsugineum* experienced a recent WGD event with a peak value between 0.7 and 0.9, corresponding to the At-α WGD event shared by all Brassicaceae species [57]. An independent WGD event was identified for *B. rapa* with a peak value of approximately 0.3, previously reported [58–60] as a Brassiceae-specific triplication (Br-α-WGD) (Supplementary Fig. 14). Therefore, *E. salsugineum* did not experience an independent WGD event after it diverged from other species, and the collinearity within the *E. salsugineum* genome corresponded to the At-α WGD event (Fig. 1b).

### Gene family and positively selected gene analyses

Furthermore, we identified the expanded and contracted gene families in our newly assembled *E. salsugineum* using CAFÉ [61] (Supplementary Tables 15 and Fig. 3b). We performed GO enrichment analysis of the expanded gene families, and the results showed that they were significantly enriched in toxin catabolic process, salt stress response, drought stress response, and signaling pathways involved in salicylic acid and gibberellin-mediated signaling (p < 0.05) (Fig. 3e; Supplementary Table 16). These gene families may be related to the environmental adaptation of *E. salsugineum*. We also identified 13 positively selected genes from the *E. salsugineum* genome (Supplementary Table 17). These genes are mainly related to ubiquitination cell growth or the salt stress pathway (Supplementary Tables 18 and 19; Supplementary Fig. 15). For example, *TOR1L5* is mainly involved in microtubule binding, *PUB3* acts as an E3 ubiquitin ligase, and the *VIP3* gene is involved in histone modification and flowering time regulation (Supplementary Table 17).

### Karyotype analysis

Karyotypes can be used to understand the relationship between chromosome changes and plant phylogeny. Schranz et al. used comparative chromosome painting (CCP) techniques to construct the ancestral karyotype (AK) model based on the *A. thaliana* genome [62, 63]. Most Brassicaceae species evolved from the ancestral Crucifer karyotype (ACK) [64] structure based on the improvement of the AK model, which consists of 8 chromosomes and 24 conserved genomic blocks (GBs, marked from A to X). These GBs were further updated to 22 GBs by merging K and L into K-L and M and N into M-N based on the *A. thaliana* genome [65] (Supplementary Table 20).

However, among six tribes (Calepineae, Coluteocarpeae, Conringieae, Eutremeae, Isatideae, and Sisymbrieae; *n* = 7) of expanded Lineage II of the family Brassicaceae, there are new GBs connections and chromosomal translocations on two AK chromosomes (Proto-Calepineae Karyotype, PCK; *n* = 7) [15, 62, 65–67]. Although three tribes (Calepineae, Coluteocarpeae and Conringieae) retain the PCK structure, there is an additional whole-arm translocation in the genomic structure of Eutremeae, Isatideae and Sisymbrieae, which is called ancestral translocation Proto-Calepineae Karyotype (tPCK; *n* = 7) [15, 65, 67]. At present, three ancestral karyotypes of Brassicaceae (ACK, PCK and tPCK) have been determined and used for chromosome structure analysis through a series of experiments and studies. ACK was considered to be the oldest among the three karyotypes, and PCK and tPCK may have evolved from ACK (Supplementary Fig. 16).

Because *E. salsugineum* belongs to the tribe Eutremeae, we chose tPCK as a reference structure in karyotype analysis. Using LAST [30] and MCScanX [68] software, we constructed a genome-wide dot plot between *E. salsugineum* and *A. thaliana* and compared the seven pseudochromosomes of *E. salsugineum* with the *A. thaliana* genome to identify syntenic relationships (Supplementary Fig. 17). Then, we constructed and visualized the order and orientation of the updated 22 GBs along the seven pseudochromosomes of the *E. salsugineum* genome (Fig. 1b). We found that the genome of *E. salsugineum* has good collinearity in each GB compared with the *A. thaliana* genome and is consistent with the tPCK structure in both order and orientation (Supplementary Fig. 14). Therefore, the *E. salsugineum* karyotype is very conservative.

## Discussion

The availability of high-quality reference genome sequences has been essential in evolutionary biology, genetics, and biodiversity conservation. Combining data from the Hi-C, Nanopore, and Illumina platforms to guide genome sequence assemblies has proven to be an effective method to improve assembly quality. Recently, several high-quality assemblies of reference genomes were generated through the integration of long reads and Hi-C data [21, 22, 69]. The long reads lead to low fragmentation in the repeat-rich region. Therefore, this integrated genome assembly method yielded a significantly improved *E. salsugineum* reference genome in the form of a smaller subset of molecules ordered and oriented into seven chromosome-scale pseudomolecules.

In recent years, revolutionary advances in DNA sequencing technologies have dramatically accelerated plant genome research [23, 70–73]. However, genome assemblies obtained solely from assembling short reads obtained by shotgun sequencing remain in a ‘draft’ stage characterized by unordered contigs or scaffolds of variable and often poor quality in repeat-rich regions. A reference-quality genome sequence is essential for variant identification [74] and facilitates revealing plant-specific traits [69, 75]. Assisted by the Nanopore long read and the Hi-C data, the quality of the previously sequenced *E. salsugineum* genome [12, 13] was greatly improved, especially in the repeat-rich regions. Although the pseudomolecules consist of a small number of large scaffolds, they represent the majority of the genome data and cover 97.5% of all annotated genes. Pseudomolecules provide information on the distribution of DNA elements along genomic regions, such as transposable elements, tandem repeats, and functional genes. Furthermore, karyotype analysis of *E. salsugineum* not only suggested that the chromosome structure of this species was very conservative and consistent with the tPCK structure with respect to both order and orientation but also confirmed that there was no independent whole-genome duplication event for *E. salsugineum* after its split from other species.

Compared with two previous genome assemblies, this new assembly has great improvements in genome size (295.5 Mb), contig N50 size (3.05 Mb) and scaffold N50 size (36.82 Mb). Our EsaV3 assembly captured more sequences and showed less fragmentation than both previous genome versions. This genome contains more transposable elements (156.1 Mb; 52.83% vs. 51.78% and 51.40%), and the LTR is the major contributor, comprising 22.08% of the total genome size. We further found that there was an obvious LTR insertion recently occurring in the *E. salsugineum* genome, and the genes near the location of recent LTR insertion were significantly enriched in DNA integration and enzyme activities. The number of annotated gene models in our assembly were comparable to two previous versions, and the differences between them might be due to the lower fragmented genes with higher mean gene length and exons per gene in our assembly (Table 1) [76]. The improved assembly also enable us to discover features such as improved gene prediction, complete catalog of repeats, improved reconstruction of karyotype, and better understanding of genome evolution and structure.

## Conclusions

We assembled a new high-quality genome for *E. salsugineum* based on Nanopore long reads and chromosome conformation capture data. This new assembly is more contiguous in complex regions, providing a valuable foundation for comparative genomic studies and serving as a new reference for the functional analysis of plant abiotic stress tolerance in plants.

## Materials and methods

### Plant materials, DNA extraction and genome sequencing

Seeds of *E. salsugineum* were collected in the Yellow River Delta area, Shandong province, China (37°26′ N, 118°04′ E) and germinated in the greenhouse. It is permitted to collect some plant samples for scientifc researches based on Regulations on the Protection of Wild Plants of the People’s Re- public of China. *E. salsugineum* used in our study was identified by Prof. Ihsan A. Al-Shehbaz and Guoqian Hao according to the morphological characteristics and the voucher specimen was deposited in Herbarium of Sichuan University (Ljq-hao14-E07_SZ) [77].

We extracted high-quality genomic DNA from fresh young leaves of a 4-week-old soil-grown *E. salsugineum* (Shandong ecotype) plant cultivated in the greenhouse using the cetyltrimethylammonium bromide (CTAB) method [78].

For Oxford Nanopore long-read sequencing, we constructed DNA libraries and sequenced the reads using a Nanopore GridION X5 sequencer. Then, we removed sequencing adapters and filtered reads with low quality and short lengths. This yielded a total of 19.40 Gb of data and 17.57 Gb of clean sequencing data with an N50 of 28.4 kb (Supplementary Table 2). For Illumina sequencing, we prepared a paired-end library with insert sizes of 500 bp and subsequently sequenced the reads on an Illumina HiSeqX-Ten platform for error correction and K-mer analysis. This generated 18.06 Gb of clean Illumina data (Supplementary Table 2).

### Genome assembly and pseudochromosome construction

For genome assembly, we first used Canu [24] to independently assemble the high-quality Nanopore subreads and yielded 297.1 Mb assemblies, with contig N50 values of 3.1 Mb and a contig number of 1,244. Then, we found that 16 contigs showed extraordinarily high GC content (> 0.6) (Supplementary Fig. 2). We removed the polluted area and corrected the assembled contigs in the Nanopore reads by using paired-end Illumina short reads by Pilon v.1.13 [25]. Finally, we obtained a 295.1 Mb genome assembly with a contig N50 of 3.1 Mb. The genome contained 1,228 contigs, and the longest contig was 17.44 Mb with 37.56% GC content. These contigs were further anchored to chromosomes by the Hi-C technique.

We ground ~ 3 g of fresh young leaves of the same *E. salsugineum* accession into powder in liquid nitrogen for Hi-C experiments and constructed a Hi-C library following Louwers et al [79]. We first fixed the leaves with formaldehyde and digested the cross-linked DNAs with Dpn II. After DNA ligation, purification, and fragmentation, the raw reads were generated by the Illumina HiSeqX Ten platform. After quality control using fastp v0.12.648, we obtained a total of 39.51 Gb of clean reads for Hi-C analyses.

We first performed a preassembly by splitting contigs into segments of 150 kb on average and mapping the Hi-C data to the contigs using bowtie2 v.2.3.2 [80] with the parameters “--very -sensitive -L 30” to correct contig errors. Scaffolding of the draft assembly with Hi-C data was performed with the 3D-DNA pipeline [26]. Briefly, clean Hi-C reads were mapped to the assembly and then a candidate chromosome-length assembly was generated by the pipeline after correcting for misjoins, ordering, orienting, and anchoring contigs from the draft assembly. The 3D-DNA pipeline was run with the following parameters: --editor-repeat-coverage 10. Then, we clustered and reordered the corrected scaffolds into pseudochromosomes. We finally adjusted the order and direction of the scaffolds on the pseudochromosomes by visualizing their interactions in the Hi-C heatmap with a resolution set at 200 kb in the Juicebox Assembly Tools (JBAT) [27]. To evaluate the completeness and quality of the final assembled genome, we applied a BUSCO v.5.2.2 [28] test using gene content from the Embryophyta_odb10 database. To further evaluate the consensus quality value and the completeness of the assembled genome, we used Merqury [29] software by preparing meryl dbs and conducting overall assembly evaluation.

### Repeat annotation

We employed a strategy that combined homology alignment and de novo searches to identify repetitive elements. The RepeatModeler v.1.0.11 [81] with RECON and RepeatScout were used to predict de novo transposable elements (TEs). Following this, the RepeatMasker v.4.0.7 [82] was then used to annotate repeats with the ab initio repeat database and Repbase (20.05). Finally, We combined the identified repeats as the final annotated results.

We used LTRharvest v1.5.10 [83] (parameters: -similar 90 -vic 10 -seed 20 -seqids yes -minlenltr 100 -maxlenltr 7000 -mintsd 4 -maxtsd 6 -motif TGCA -motifmis 1) and LTR_Finder v1.06 [84] (parameters: -D 15,000 -d 1000 -L 7000 -l 100 -p 20 -C -M 0.9) to identify candidate LTR-RTs. We used LTR_retriever v1.9 [85] software to integrate previous results of LTR_Finder and LTRharvest and filter out the false positive LTR-RTs. Then, we estimated the insertion times of LTR-RTs in three related Brassicaceae species (*A. thaliana*, *E. heterophyllum* and *E. salsugineum*) based on T = K/2r (K: divergence rate; r: neutral mutation rate, 7 × 10^–9^/site/year).

### Gene prediction and functional annotation

To improve the accuracy of annotation as much as possible, we combined transcriptome-based, homology-based, and de novo predictions to predict the protein-coding genes of the *E. salsugineum* genome. During the transcriptome-based prediction, we downloaded the previously published RNA-Seq data of this species (BioProject: PRJNA483528) from the NCBI SRA database [86] and further assembled the reads into transcripts using Trinity v2.6.6 [87] in ab initio and genome-guide mode. We then run the Assemble Spliced Alignment (PASA) v.2.1.0 [88] analysis process to align the transcripts to the *E. salsugineum* genome to carry out open reading frame (ORF) and protein coding gene prediction. For de novo prediction, we used Augustus v3.3.1 [89] with parameters trained using PASA self-trained gene models and GlimmerHMM v3.0.4 [90] to annotate the genome sequence of *E. salsugineum*. For homology-based prediction, we downloaded seven sequenced protein sequences of *A. thaliana* [32], *C. rubella* [47], *B. rapa* [48], *S. parvula* [49], *Raphanus raphanistrum* [51], *E. heterophyllum* [33] and *E. yunnanense* [33] and aligned them against the *E. salsugineum* genome using TBLASTN v.2.6.0 [91] (e-value cutoff 1e − 5). After filtering low-quality results, we used Exonerate v2.2.0 to predict the gene structure. We used EVidenceModeler (EVM) v1.1.1 [92] software to integrate the results of the above three annotation methods and obtained the final protein-coding gene set.

We annotated the functions of the predicted genes using BLASTP v.2.6.0 [91] (E-value cutoff 1e − 5) based on entries in the Swiss-Prot and TrEMBL databases. Protein motifs and domains were identified by searching against InterPro. The functions and pathways of the genes were determined according to the GO and KEGG databases by using Blast2GO [93]. Finally, a total of 24,448 protein-coding genes (96.26%) were successfully annotated through reference to one or more databases.

### Whole-genome alignment of *E. salsugineum* genomes

We aligned our EsaV3 genome to the two previously reported genome sequences of *E. salsugineum* by LAST [30], following a five-step procedure (https://github.com/mcfrith/last-genome-alignments) and then verified the alignment results using in-house Perl scripts.

### Phylogenetic and gene family analyses

We used protein-coding genes of *E. salsugineum* and nine other Brassicaceae species *(A. thaliana* [32], *C. rubella* [47], *B. rapa* [48], *S. parvula* [49], *Isatis indigotica* [50], *R. raphanistrum* [51], *E. heterophyllum* [33] and *E. yunnanense* [33]) with *A. arabicum* [46] as an outgroup for gene family clustering analysis. To remove the interference caused by alternative splicing, the longest transcript was selected as a reference for downstream analyses. Single-copy orthologous genes were identified across *E. salsugineum* and the other nine species using OrthoFinder v2.3.12 [52] and aligned using MAFFT v.7.313 [94]. Then, we used the PROTGAMMALGX model of RAxML v8.0.0 [95] software to construct a phylogenetic tree with the tandem protein sequences as input. We estimated the divergence time of each branch using the MCMCTREE program of PAML v.4.9 [53] with the ‘correlated molecular clock’ model. We used the estimated divergence time in the TimeTree database (http://www.timetree.org/) for (1) *A. thaliana*-*C. rubella* (7.4–12.8 Mya), (2) *R. raphanistrum*-*B. rapa* (2.2–9.8 Mya) and (3) *A. thaliana*-*A. arabicum* (32–43 Mya) to calibrate the constructed phylogenetic tree.

Gene families that had undergone expansion or contraction were identified in the ten sequenced species using CAFÉ and the previously constructed phylogenetic tree. We extracted the significantly expanded gene families (P < 0.05) and performed enrichment analysis for these genes using Blast2GO based on the results of functional annotations.

### Synteny and whole-genome duplication

We identified syntenic blocks within the genomes of *E. salsugineum, A. thaliana* [32], *B. rapa* [48] and *I. indigotica* [50] by applying MCScanX software with default parameters. Then, we used the in-house Perl script “add_ka_and_ks_to_collinearity.pl” in the MCScanX package to calculate the synonymous substitution rates (Ks) values between collinear gene pairs among these four genomes to identify WGD events. We also converted the Ks values into divergence times based on the formula T = Ks/2r (T: divergence time; r: neutral substitution rate, 7 × 10^–9^/site/year).

### Identification of positively selected genes

We detected positively selected genes in the single-copy orthologous gene families among the genomes of ten Brassicaceae species (*A. thaliana* [32], *A. arabicum* [46], *C. rubella* [47], *B. rapa* [48], *S. parvula* [49], *I. indigotica* [50], *R. raphanistrum* [51], *E. salsugineum, E. heterophyllum* [33] and *E. yunnanense* [33]) using Proteinortho v6.0.2 [96] with the protein coding genes of these ten genomes as input files. PosiGene v0.1 [97] was used to perform whole-genome detection of the positively selected genes (PSGs) with *E. salsugineum* as the foreground branch. Based on the P value cutoff of 0.05 after false discovery rate (FDR) correction, we identified the PSGs in *E. salsugineum* and performed functional enrichment analysis for PSGs.

## Electronic supplementary material

Below is the link to the electronic supplementary material.

Supplementary Material 1

## Data Availability

Raw sequencing reads used for de novo whole-genome assembly and the final genome have been deposited at the NCBI under the BioProject PRJNA577380.
