# Supplementary material for: A high-quality chromosome-level Eutrema salsugineum genome, an extremophile plant model
Source: BMC Genomics. 2023 Apr 5;24:174. doi: 10.1186/s12864-023-09256-x (PMC10077641; doi:10.1186/s12864-023-09256-x)
Supplement: Supplementary file 1 — Supplementary Material 1 [file 12864_2023_9256_MOESM1_ESM.docx]

**Supplementary data**

Supplementary Table 1 Summary of sequencing data for *E. salsugineum* genome

| Pair-end libraries | Number of reads | Total library size | Read length/ N50 | Estimated coverage |
| --- | --- | --- | --- | --- |
| Nanopore | 891,987 | 17.57 Gb | 28.36 kb | 62.75× |
| Illumina | 60,202,319 | 18.06 Gb | 150 bp | 64.5× |
| Hi-C | 131,683,866 | 39.51 Gb | 150 bp | 141.09× |

Supplementary Table 2 Preliminary assembly results of *E. salsugineum* genome

| Stat Type | Preliminary Assembly | | | Polish Genome | | | |
| --- | --- | --- | --- | --- | --- | --- | --- |
|  | Contig Length（bp） | Contig Number | | Contig Length（bp） | | Contig Number | |
| N50 | 3,113,161 | | 28 | | 3,113,161 | | 27 |
| N60 | 2,026,629 | | 39 | | 2,026,629 | | 38 |
| N70 | 1,268,957 | | 58 | | 1,268,957 | | 56 |
| N80 | 480,876 | | 93 | | 436,014 | | 93 |
| N90 | 52,295 | | 438 | | 52,100 | | 437 |
| Longest | 17,436,329 | | 1 | | 17,436,329 | | 1 |
| Total | 300,346,232 | | 1,244 | | 295,082,356 | | 1,228 |
| Length>=1kb | 300,346,232 | | 1,244 | | 295,082,356 | | 1,228 |
| Length>=2kb | 300,346,232 | | 1,244 | | 295,082,356 | | 1,228 |
| Length>=5kb | 300,333,762 | | 1,241 | | 295,069,886 | | 1,225 |

Supplementary Table 3 Statistics for genome assembly of *E. salsugineum*

| Genome feature | Value |
| --- | --- |
| Total scaffold number | 655 |
| Total length (bp) | 295,494,356 |
| Longest scaffold length (bp) | 42,208,000 |
| Counts of scaffold N50 | 4 |
| Scaffold N50 length (bp) | 36,815,000 |
| Counts of scaffold N90 | 7 |
| Scaffold N90 length (bp) | 35,294,615 |
| Number of contigs | 1,479 |
| Longest contig length (bp) | 17,436,329 |
| Counts of contig N50 | 29 |
| Contig N50 length (bp) | 3.053,001 |
| Counts of contig N90 | 458 |
| Contig N90 length (bp) | 48,467 |
| GC content (%) | 0.3756 |
| Gap (%) | 0.139 |

Supplementary Table 4 Summary gene completeness (BUSCO) of *E. salsugineum* genome

| Parameter | BUSCO groups（%） |
| --- | --- |
| Complete | 99.1 % |
| Single | 97.6 % |
| Duplicated | 1.5 % |
| Fragmented | 0.2 % |
| Missing | 0.7 % |
| Total | 100.0 % |

**Supplementary Table 5 Repeat content within the genome of *E. salsugineum***

| Types | Length (bp) | % of genome |
| --- | --- | --- |
| DNA transposons | 8,791,226 | 2.97 |
| LTRs | 65,284,740 | 22.09 |
| LINEs | 4,317,146 | 1.46 |
| SINEs | 82,734 | 0.02 |
| Simple repeat | 3,151,820 | 1.06 |
| Satellite | 1,551,738 | 0.52 |
| Low complexity | 1,058,926 | 0.35 |
| Rolling-circles | 3,039,443 | 1.02 |
| Unknown | 75,465,909 | 25.53 |
| Total | 156,124,645 | 52.83 |

**S****upplementary Table 6 Classification of LTR-RTs in the genomes of *E. salsugineum* and other two species**

| LTR-RTs | % of genome | | |
| --- | --- | --- | --- |
|  | *E. salsugineum* | *E. heterophyllum* | *A. thaliana* |
| *Gypsy* | 14.20 | 11.06 | 5.09 |
| *Copia* | 5.82 | 3.84 | 0.46 |
| Unknown | 2.07 | 0.27 | 1.63 |
| Total | 22.09 | 15.17 | 7.18 |

**Supplementary Table 7 Classification of LTR/Gypsy and LTR/Copia in the genomes of *E. salsugineum* and *E. heterophyllum***

| LTR/Gypsy | Sequence number | | |
| --- | --- | --- | --- |
|  | *E. salsugineum* | *E. heterophyllum* | |
| Athila | 79 | | 2 |
| CRM | 97 | | 43 |
| Galadriel | 1 | | 0 |
| Reina | 43 | | 42 |
| Retand | 79 | | 12 |
| Tekay | 4 | | 8 |
| Total | 303 | | 107 |

| LTR/Copia | Sequence number | | |
| --- | --- | --- | --- |
|  | *E. salsugineum* | *E. heterophyllum* | |
| Ale | 123 | | 109 |
| Angela | 4 | | 0 |
| Bianca | 11 | | 5 |
| Ikeros | 7 | | 2 |
| Ivana | 55 | | 41 |
| SIRE | 23 | | 4 |
| TAR | 4 | | 8 |
| Tork | 63 | | 35 |
| Total | 290 | | 204 |

Supplementary Table 8 Statistics of LTR-RTs between *E. salsugineum* and *E. heterophyllum*

| Species | LTR-RTs size (bp) | specific LTR-RTs (bp) | non-specific LTR-RTs (bp) |
| --- | --- | --- | --- |
| *E. salsugineum* | 65,284,740 | 41,110,336 | 24,174,404 |
| *E. heterophyllum* | 52,999,244 | 33,468,741 | 19,530,502 |

Supplementary Table 9 The LTR-RTs content of *E. salsugineum* and other two species

| Species | Mean LTRs size (bp) | Number |
| --- | --- | --- |
| *E. salsugineum* | 8,714.54 | 2125 |
| *E. heterophyllum* | 6,961.44 | 878 |
| *A. thaliana* | 6,485.32 | 170 |

**Supplementary Table 10 Summary of the predicted protein-coding genes**

| Genome version | EsaV3 (this study) |
| --- | --- |
| Total genes | 25,399 |
| Total exon | 136,411 |
| Mean exon number per gene | 5.37 |
| Mean gene length (bp) | 2,559 |
| Mean exon length (bp) | 295 |
| Mean intron length (bp) | 223 |

**Supplementary Table 11 Identified genes near the location of recent LTR insertion in *E. salsugineum***

| Query.Chr | q.start | q.end | Subjiect.Chr | s.start | s.end | Gene.ID |
| --- | --- | --- | --- | --- | --- | --- |
| Chr3 | 10471446 | 10475686 | Chr3 | 10475641 | 10475958 | Esa07733.t1 |
| Chr3 | 1245529 | 1255867 | Chr3 | 1255855 | 1260363 | Esa07164.t1 |
| Chr3 | 10381987 | 10391351 | Chr3 | 10379702 | 10382048 | Esa07730.t1 |
| Chr3 | 10600603 | 10620489 | Chr3 | 10615104 | 10616495 | Esa07736.t1 |
| Chr3 | 10600603 | 10620489 | Chr3 | 10616510 | 10617082 | Esa07737.t1 |
| Chr4 | 10560135 | 10564513 | Chr4 | 10560394 | 10560840 | Esa12220.t1 |
| Chr4 | 10560135 | 10564513 | Chr4 | 10560856 | 10562493 | Esa12221.t1 |
| Chr4 | 10560135 | 10564513 | Chr4 | 10562530 | 10563853 | Esa12222.t1 |
| Chr4 | 11963112 | 11969922 | Chr4 | 11963825 | 11967842 | Esa12404.t1 |
| Chr4 | 11963112 | 11969922 | Chr4 | 11968116 | 11968637 | Esa12405.t1 |
| Chr4 | 11963112 | 11969922 | Chr4 | 11969694 | 11970578 | Esa12406.t1 |
| Chr5 | 20276142 | 20279643 | Chr5 | 20277744 | 20278094 | Esa14822.t1 |
| Chr5 | 20276142 | 20279643 | Chr5 | 20278130 | 20279003 | Esa14823.t1 |
| Chr5 | 20276142 | 20279643 | Chr5 | 20279308 | 20279808 | Esa14824.t1 |
| Chr5 | 9852874 | 9862484 | Chr5 | 9852358 | 9852876 | Esa14640.t1 |
| Chr5 | 10249897 | 10253989 | Chr5 | 10250528 | 10252219 | Esa14657.t1 |
| Chr5 | 3463806 | 3471312 | Chr5 | 3450624 | 3493476 | Esa14286.t1 |
| Chr5 | 18474612 | 18485590 | Chr5 | 18323385 | 18668059 | Esa14771.t1 |
| Chr6 | 14738840 | 14745784 | Chr6 | 14738475 | 14741287 | Esa20086.t1 |
| Chr6 | 14738840 | 14745784 | Chr6 | 14742021 | 14746177 | Esa20087.t1 |
| Chr6 | 11234760 | 11245014 | Chr6 | 11241417 | 11241807 | Esa19720.t1 |
| Chr6 | 11234760 | 11245014 | Chr6 | 11242441 | 11244437 | Esa19721.t1 |
| Chr6 | 34472349 | 34484348 | Chr6 | 34468346 | 34540917 | Esa20685.t1 |
| Chr6 | 14986518 | 14994058 | Chr6 | 14993571 | 14993804 | Esa20102.t1 |
| Chr6 | 14986518 | 14994058 | Chr6 | 14989718 | 14992321 | Esa20101.t1 |
| Chr7 | 27581261 | 27586428 | Chr7 | 27581632 | 27583023 | Esa22669.t1 |
| Chr7 | 27581261 | 27586428 | Chr7 | 27583092 | 27586063 | Esa22670.t1 |
| Chr7 | 8729362 | 8746786 | Chr7 | 8729616 | 8730058 | Esa22168.t1 |
| Chr7 | 28270781 | 28281348 | Chr7 | 28271471 | 28271788 | Esa22721.t1 |

**Supplementary Table 12 GO enrichment results of** **genes near the location of recent LTR insertion in *E. salsugineum***

| GO.ID | Term | GeneRatio | BgRatio | P-value | FDR |
| --- | --- | --- | --- | --- | --- |
| GO:0015074 | DNA integration | 4 | 188 | 3.75E-07 | 2.43E-05 |
| GO:0003964 | RNA-directed DNA polymerase activity | 4 | 219 | 3.08E-06 | 8.46E-05 |
| GO:0034061 | DNA polymerase activity | 4 | 238 | 4.28E-06 | 8.46E-05 |
| GO:0004190 | aspartic-type endopeptidase activity | 4 | 254 | 5.54E-06 | 8.46E-05 |
| GO:0070001 | aspartic-type peptidase activity | 4 | 254 | 5.54E-06 | 8.46E-05 |
| GO:0004519 | endonuclease activity | 4 | 332 | 1.60E-05 | 1.95E-04 |
| GO:0016779 | nucleotidyltransferase activity | 4 | 364 | 2.30E-05 | 2.34E-04 |
| GO:0004518 | nuclease activity | 4 | 456 | 5.57E-05 | 4.85E-04 |
| GO:0004175 | endopeptidase activity | 4 | 514 | 8.89E-05 | 6.78E-04 |
| GO:0006259 | DNA metabolic process | 4 | 813 | 1.25E-04 | 4.07E-03 |
| GO:0070011 | peptidase activity, acting on L-amino acid peptides | 4 | 665 | 2.41E-04 | 1.64E-03 |
| GO:0008233 | peptidase activity | 4 | 684 | 2.69E-04 | 1.64E-03 |
| GO:0016772 | transferase activity, transferring phosphorus-containing groups | 5 | 1384 | 3.07E-04 | 1.70E-03 |
| GO:0016788 | hydrolase activity, acting on ester bonds | 4 | 1073 | 1.50E-03 | 7.62E-03 |
| GO:0004103 | choline kinase activity | 1 | 4 | 2.15E-03 | 1.01E-02 |
| GO:0006310 | DNA recombination | 2 | 264 | 4.56E-03 | 9.87E-02 |
| GO:0000095 | S-adenosyl-L-methionine transmembrane transporter activity | 1 | 13 | 6.98E-03 | 3.04E-02 |
| GO:0051185 | coenzyme transporter activity | 1 | 15 | 8.05E-03 | 3.07E-02 |
| GO:0072349 | modified amino acid transmembrane transporter activity | 1 | 15 | 8.05E-03 | 3.07E-02 |
| GO:0007130 | synaptonemal complex assembly | 1 | 24 | 9.72E-03 | 1.26E-01 |
| GO:0070193 | synaptonemal complex organization | 1 | 24 | 9.72E-03 | 1.26E-01 |
| GO:0051184 | cofactor transporter activity | 1 | 22 | 1.18E-02 | 4.23E-02 |
| GO:0016787 | hydrolase activity | 5 | 3142 | 1.33E-02 | 4.51E-02 |
| GO:1901682 | sulfur compound transmembrane transporter activity | 1 | 33 | 1.76E-02 | 5.66E-02 |
| GO:0007129 | synapsis | 1 | 45 | 1.82E-02 | 1.87E-01 |
| GO:0016740 | transferase activity | 5 | 3435 | 1.96E-02 | 5.97E-02 |
| GO:0045143 | homologous chromosome segregation | 1 | 50 | 2.02E-02 | 1.87E-01 |
| GO:0015211 | purine nucleoside transmembrane transporter activity | 1 | 48 | 2.56E-02 | 7.43E-02 |
| GO:0070192 | chromosome organization involved in meiotic cell cycle | 1 | 70 | 2.81E-02 | 2.15E-01 |
| GO:0005337 | nucleoside transmembrane transporter activity | 1 | 55 | 2.93E-02 | 8.11E-02 |
| GO:0090304 | nucleic acid metabolic process | 4 | 3456 | 2.98E-02 | 2.15E-01 |
| GO:0045132 | meiotic chromosome segregation | 1 | 84 | 3.37E-02 | 2.19E-01 |
| GO:0006139 | nucleobase-containing compound metabolic process | 4 | 3850 | 4.35E-02 | 2.46E-01 |
| GO:0007127 | meiosis I | 1 | 114 | 4.55E-02 | 2.46E-01 |
| GO:0015932 | nucleobase-containing compound transmembrane transporter activity | 1 | 87 | 4.59E-02 | 1.22E-01 |
| GO:1901505 | carbohydrate derivative transporter activity | 1 | 94 | 4.95E-02 | 1.24E-01 |
| GO:0008270 | zinc ion binding | 2 | 696 | 5.09E-02 | 1.24E-01 |

**Supplementary Table 13 GO enrichment results of 1,153 newly annotated genes in *E. salsugineum***

| Class | GO.ID | Term | Annotated | Significant | Classic  Fisher |
| --- | --- | --- | --- | --- | --- |
| BP | GO:0015074 | DNA integration | 188 | 23 | 3.40E-14 |
| BP | GO:0032196 | transposition | 15 | 9 | 2.80E-13 |
| BP | GO:0000278 | mitotic cell cycle | 248 | 11 | 6.00E-09 |
| BP | GO:0006310 | DNA recombination | 264 | 16 | 5.70E-06 |
| BP | GO:0044419 | interspecies interaction between organisms | 1447 | 25 | 8.00E-05 |
| BP | GO:0006418 | tRNA aminoacylation for protein translation | 72 | 7 | 0.00015 |
| BP | GO:0000226 | microtubule cytoskeleton organization | 180 | 11 | 0.00016 |
| BP | GO:0009772 | photosynthetic electron transport in photosystem II | 13 | 3 | 0.00104 |
| BP | GO:0015937 | coenzyme A biosynthetic process | 14 | 3 | 0.0013 |
| BP | GO:0009109 | coenzyme catabolic process | 15 | 3 | 0.00161 |
| BP | GO:0009166 | nucleotide catabolic process | 16 | 3 | 0.00196 |
| BP | GO:0015986 | ATP synthesis coupled proton transport | 17 | 3 | 0.00235 |
| BP | GO:0072523 | purine-containing compound catabolic process | 17 | 3 | 0.00235 |
| BP | GO:0006611 | protein export from nucleus | 45 | 3 | 0.00248 |
| BP | GO:0009407 | toxin catabolic process | 90 | 6 | 0.00325 |
| BP | GO:0090354 | regulation of auxin metabolic process | 22 | 3 | 0.00366 |
| BP | GO:0006637 | acyl-CoA metabolic process | 50 | 4 | 0.00435 |
| BP | GO:0048268 | clathrin coat assembly | 21 | 3 | 0.00438 |
| BP | GO:0072583 | clathrin-dependent endocytosis | 43 | 4 | 0.00488 |
| BP | GO:0019684 | photosynthesis, light reaction | 134 | 9 | 0.00748 |
| BP | GO:0006749 | glutathione metabolic process | 108 | 6 | 0.00787 |
| BP | GO:0031640 | killing of cells of other organism | 26 | 3 | 0.00807 |
| BP | GO:0031126 | snoRNA 3'-end processing | 20 | 3 | 0.00851 |
| BP | GO:0006412 | translation | 611 | 21 | 0.00885 |
| BP | GO:0006166 | purine ribonucleoside salvage | 10 | 2 | 0.0106 |
| BP | GO:0044209 | AMP salvage | 10 | 2 | 0.0106 |
| BP | GO:0031120 | snRNA pseudouridine synthesis | 10 | 2 | 0.0106 |
| BP | GO:0048544 | recognition of pollen | 55 | 4 | 0.01163 |
| BP | GO:0006351 | transcription, DNA-templated | 1801 | 21 | 0.01458 |
| BP | GO:0018298 | protein-chromophore linkage | 66 | 4 | 0.02145 |
| BP | GO:1901136 | carbohydrate derivative catabolic process | 68 | 4 | 0.02365 |
| BP | GO:0031118 | rRNA pseudouridine synthesis | 17 | 2 | 0.02975 |
| BP | GO:0071230 | cellular response to amino acid stimulus | 18 | 2 | 0.03312 |
| BP | GO:0031936 | negative regulation of chromatin silencing | 18 | 2 | 0.03312 |
| BP | GO:0016024 | CDP-diacylglycerol biosynthetic process | 19 | 2 | 0.03664 |
| BP | GO:0006816 | calcium ion transport | 52 | 4 | 0.04742 |
| BP | GO:0007186 | G protein-coupled receptor signaling pathway | 22 | 2 | 0.04797 |
| CC | GO:0045263 | proton-transporting ATP synthase complex, coupling factor F(o) | 12 | 5 | 9.40E-07 |
| CC | GO:0005739 | mitochondrion | 1723 | 51 | 2.90E-06 |
| CC | GO:0005874 | microtubule | 258 | 15 | 3.30E-05 |
| CC | GO:0033643 | host cell part | 11 | 3 | 0.0007 |
| CC | GO:0015934 | large ribosomal subunit | 153 | 6 | 0.00075 |
| CC | GO:0005905 | clathrin-coated pit | 35 | 4 | 0.00273 |
| CC | GO:0009535 | chloroplast thylakoid membrane | 418 | 15 | 0.00494 |
| CC | GO:0034399 | nuclear periphery | 25 | 3 | 0.00825 |
| CC | GO:0005763 | mitochondrial small ribosomal subunit | 10 | 2 | 0.01162 |
| CC | GO:0005834 | heterotrimeric G-protein complex | 11 | 2 | 0.01405 |
| CC | GO:0005687 | U4 snRNP | 11 | 2 | 0.01405 |
| CC | GO:0031429 | box H/ACA snoRNP complex | 12 | 2 | 0.01667 |
| CC | GO:0005753 | mitochondrial proton-transporting ATP synthase complex | 15 | 2 | 0.02566 |
| CC | GO:0005871 | kinesin complex | 41 | 3 | 0.03142 |
| CC | GO:0030136 | clathrin-coated vesicle | 79 | 4 | 0.04423 |
| CC | GO:0005794 | Golgi apparatus | 1169 | 20 | 0.04997 |
| MF | GO:0004518 | nuclease activity | 448 | 55 | 1.00E-30 |
| MF | GO:0004519 | endonuclease activity | 323 | 28 | 5.80E-14 |
| MF | GO:0003964 | RNA-directed DNA polymerase activity | 219 | 25 | 9.80E-14 |
| MF | GO:0003676 | nucleic acid binding | 4221 | 100 | 2.70E-13 |
| MF | GO:0004190 | aspartic-type endopeptidase activity | 254 | 24 | 1.90E-11 |
| MF | GO:0005200 | structural constituent of cytoskeleton | 31 | 10 | 7.40E-11 |
| MF | GO:0005525 | GTP binding | 278 | 18 | 2.10E-06 |
| MF | GO:0008270 | zinc ion binding | 696 | 30 | 5.10E-06 |
| MF | GO:0048038 | quinone binding | 27 | 6 | 6.00E-06 |
| MF | GO:0016655 | oxidoreductase activity, acting on NAD(P)H, quinone or similar compound as acceptor | 53 | 7 | 8.10E-06 |
| MF | GO:0032549 | ribonucleoside binding | 290 | 22 | 3.30E-05 |
| MF | GO:0003924 | GTPase activity | 200 | 13 | 5.30E-05 |
| MF | GO:0004812 | aminoacyl-tRNA ligase activity | 72 | 7 | 0.00026 |
| MF | GO:0046872 | metal ion binding | 3823 | 96 | 0.00045 |
| MF | GO:0046933 | proton-transporting ATP synthase activity, rotational mechanism | 27 | 4 | 0.00118 |
| MF | GO:0016289 | CoA hydrolase activity | 16 | 3 | 0.00252 |
| MF | GO:0045156 | electron transporter, transferring electrons within the cyclic electron transport pathway of photosynthesis activity | 16 | 3 | 0.00252 |
| MF | GO:0003899 | DNA-directed 5'-3' RNA polymerase activity | 55 | 5 | 0.00272 |
| MF | GO:0016168 | chlorophyll binding | 38 | 4 | 0.00427 |
| MF | GO:0002161 | aminoacyl-tRNA editing activity | 21 | 3 | 0.00561 |
| MF | GO:0005545 | 1-phosphatidylinositol binding | 21 | 3 | 0.00561 |
| MF | GO:0004364 | glutathione transferase activity | 94 | 6 | 0.00618 |
| MF | GO:0032050 | clathrin heavy chain binding | 23 | 3 | 0.00728 |
| MF | GO:0019843 | rRNA binding | 135 | 7 | 0.00975 |
| MF | GO:0005516 | calmodulin binding | 245 | 10 | 0.01138 |
| MF | GO:0016462 | pyrophosphatase activity | 836 | 27 | 0.01214 |
| MF | GO:0016597 | amino acid binding | 29 | 3 | 0.01391 |
| MF | GO:0005049 | nuclear export signal receptor activity | 11 | 2 | 0.01518 |
| MF | GO:0005546 | phosphatidylinositol-4,5-bisphosphate binding | 31 | 3 | 0.01668 |
| MF | GO:0070567 | cytidylyltransferase activity | 12 | 2 | 0.01801 |
| MF | GO:0003677 | DNA binding | 2407 | 33 | 0.02009 |
| MF | GO:0043531 | ADP binding | 156 | 7 | 0.02018 |
| MF | GO:0000049 | tRNA binding | 35 | 3 | 0.02309 |
| MF | GO:0015078 | proton transmembrane transporter activity | 147 | 10 | 0.02912 |
| MF | GO:0008066 | glutamate receptor activity | 16 | 2 | 0.03127 |
| MF | GO:0051139 | metal ion:proton antiporter activity | 17 | 2 | 0.03504 |
| MF | GO:0009982 | pseudouridine synthase activity | 17 | 2 | 0.03504 |
| MF | GO:0051287 | NAD binding | 74 | 4 | 0.04095 |
| MF | GO:0004523 | RNA-DNA hybrid ribonuclease activity | 47 | 3 | 0.04914 |
| MF | GO:0004523 | RNA-DNA hybrid ribonuclease activity | 47 | 3 | 0.04914 |

**Supplementary Table 14 Results of functional annotation of *E. salsugineum* genome**

| Annotation database | Annotated number | Percent of annotated genes (%) |
| --- | --- | --- |
| Swiss-Prot | 18,824 | 74.11 |
| TrEMBL | 24,392 | 96.04 |
| InterPro | 20,037 | 78.89 |
| Gene Ontology (GO) | 17,963 | 70.72 |
| KEGG Pathway | 10,185 | 40.10 |
| Annotated | 24,448 | 96.26 |
| Unannotated | 951 | 3.74 |

Supplementary Table 15 Results of expansions and contractions of gene families

| Species | Expanded fams | Contracted fams | Rapidly evolving fams |
| --- | --- | --- | --- |
| *Capsella rubella* | 1069 | 882 | 147 |
| *Arabidopsis thaliana* | 885 | 880 | 83 |
| *Isatis indigotica* | 2131 | 3138 | 238 |
| *Brassica rapa* | 4134 | 756 | 260 |
| *Raphanus raphanistrum* | 2428 | 2652 | 280 |
| *Schrenkiella parvula* | 988 | 2621 | 100 |
| *Eutrema salsugineum* | 852 | 2588 | 153 |
| *Eutrema heterophyllum* | 1874 | 1486 | 163 |
| *Eutrema yunnanense* | 1520 | 1546 | 173 |
| *Aethionema arabicum* | 1188 | 7515 | 91 |

Supplementary Table 16 GO enrichment results of significantly expanded genes in *E. salsugineum*

| Class | GO.ID | Term | Annotated | Significant | Classic Fisher |
| --- | --- | --- | --- | --- | --- |
| BP | GO:0015074 | DNA integration | 188 | 108 | 1e-30 |
| BP | GO:0006749 | glutathione metabolic process | 108 | 38 | 1e-30 |
| BP | GO:0009407 | Toxin catabolic process | 90 | 38 | 1e-20 |
| BP | GO:0010082 | regulation of root meristem growth | 59 | 22 | 2.3e-20 |
| BP | GO:0048235 | pollen sperm cell differentiation | 68 | 22 | 8.4e-19 |
| BP | GO:0090333 | regulation of stomatal closure | 71 | 22 | 2.4e-18 |
| BP | GO:0031146 | SCF-dependent proteasomal ubiquitin-dependent protein catabolic process | 82 | 20 | 1.5e-14 |
| BP | GO:0046777 | protein autophosphorylation | 242 | 32 | 2.5e-14 |
| BP | GO:0051510 | regulation of unidimensional cell growth | 116 | 22 | 1.9e-13 |
| BP | GO:0006353 | DNA-templated transcription, termination | 47 | 15 | 4.0e-13 |
| BP | GO:0016046 | detection of fungus | 10 | 8 | 7.8e-12 |
| BP | GO:0042794 | plastid rRNA transcription | 10 | 8 | 7.8e-12 |
| BP | GO:0018874 | benzoate metabolic process | 11 | 8 | 2.8e-11 |
| BP | GO:0051923 | sulfation | 21 | 10 | 3.2e-11 |
| BP | GO:0000302 | response to reactive oxygen species | 202 | 23 | 1.9e-10 |
| BP | GO:0006310 | DNA recombination | 264 | 26 | 6.9e-10 |
| BP | GO:0016567 | protein ubiquitination | 590 | 43 | 7.4e-10 |
| BP | GO:0032196 | transposition | 15 | 8 | 1.0e-09 |
| BP | GO:0034052 | positive regulation of plant-type hypersensitive response | 19 | 8 | 1.1e-08 |
| BP | GO:2000032 | regulation of secondary shoot formation | 25 | 8 | 1.3e-07 |
| BP | GO:0010112 | regulation of systemic acquired resistance | 30 | 8 | 6.5e-07 |
| BP | GO:0000966 | RNA 5'-end processing | 11 | 5 | 4.5e-06 |
| BP | GO:0044419 | interspecies interaction between organisms | 1447 | 46 | 5.9e-06 |
| BP | GO:0009738 | abscisic acid-activated signaling pathway | 369 | 25 | 1.1e-05 |
| BP | GO:0034644 | cellular response to UV | 23 | 6 | 2.0e-05 |
| BP | GO:0019253 | reductive pentose-phosphate cycle | 27 | 6 | 5.3e-05 |
| BP | GO:0010154 | fruit development | 753 | 17 | 0.00012 |
| BP | GO:0045910 | negative regulation of DNA recombination | 38 | 6 | 0.00039 |
| BP | GO:0051301 | cell division | 487 | 23 | 0.00041 |
| BP | GO:0032502 | developmental process | 3653 | 68 | 0.00049 |
| BP | GO:0016032 | viral process | 97 | 9 | 0.00089 |
| BP | GO:0009409 | response to cold | 468 | 24 | 0.00118 |
| BP | GO:0000394 | RNA splicing, via endonucleolytic cleavage and ligation | 19 | 4 | 0.00124 |
| BP | GO:0009610 | response to a symbiotic fungus | 20 | 4 | 0.00152 |
| BP | GO:0009863 | salicylic acid-mediated signaling pathway | 89 | 8 | 0.00208 |
| BP | GO:0008380 | RNA splicing | 356 | 18 | 0.00317 |
| BP | GO:0009414 | response to water deprivation | 482 | 23 | 0.00351 |
| BP | GO:0036123 | histone H3-K9 dimethylation | 13 | 3 | 0.00402 |
| BP | GO:0009853 | photorespiration | 61 | 6 | 0.00476 |
| BP | GO:0009651 | response to salt stress | 580 | 25 | 0.00859 |
| BP | GO:0006346 | DNA methylation-dependent heterochromatin assembly | 18 | 3 | 0.01042 |
| BP | GO:0009740 | gibberellic acid-mediated signaling pathway | 95 | 7 | 0.01140 |
| BP | GO:0000963 | mitochondrial RNA processing | 23 | 3 | 0.02056 |
| BP | GO:0051707 | response to other organism | 1361 | 33 | 0.02233 |
| BP | GO:0071902 | positive regulation of protein serine/threonine kinase activity | 10 | 2 | 0.02604 |
| BP | GO:2000071 | regulation of defense response by callose deposition | 10 | 2 | 0.02604 |
| BP | GO:0043405 | regulation of MAP kinase activity | 11 | 2 | 0.03129 |
| BP | GO:0032259 | methylation | 361 | 11 | 0.03199 |
| BP | GO:0002229 | defense response to oomycetes | 93 | 6 | 0.03308 |
| BP | GO:1902533 | positive regulation of intracellular signal transduction | 13 | 2 | 0.0429 |
| CC | GO:0005739 | mitochondrion | 1723 | 75 | 8.90E-11 |
| CC | GO:0009536 | plastid | 2793 | 78 | 7.90E-09 |
| CC | GO:0019005 | SCF ubiquitin ligase complex | 71 | 11 | 8.10E-07 |
| CC | GO:0005634 | nucleus | 5925 | 173 | 1.70E-05 |
| CC | GO:0045261 | proton-transporting ATP synthase complex, catalytic core F(1) | 22 | 5 | 0.00014 |
| CC | GO:0016592 | mediator complex | 72 | 8 | 0.00029 |
| CC | GO:0071004 | U2-type prespliceosome | 27 | 5 | 0.00038 |
| CC | GO:0005686 | U2 snRNP | 34 | 5 | 0.00115 |
| CC | GO:0033643 | host cell part | 11 | 3 | 0.00189 |
| CC | GO:0071013 | catalytic step 2 spliceosome | 48 | 5 | 0.00538 |
| CC | GO:0000792 | heterochromatin | 20 | 3 | 0.01113 |
| CC | GO:0005819 | spindle | 103 | 7 | 0.01121 |
| CC | GO:0009524 | phragmoplast | 113 | 7 | 0.01793 |
| MF | GO:0003676 | nucleic acid binding | 4221 | 205 | 1.00E-30 |
| MF | GO:0003964 | RNA-directed DNA polymerase activity | 219 | 114 | 1.00E-30 |
| MF | GO:0004519 | endonuclease activity | 323 | 134 | 1.00E-30 |
| MF | GO:0004190 | aspartic-type endopeptidase activity | 254 | 108 | 1.00E-30 |
| MF | GO:0008270 | zinc ion binding | 696 | 100 | 1.00E-30 |
| MF | GO:0004364 | glutathione transferase activity | 94 | 38 | 1.00E-30 |
| MF | GO:0004709 | MAP kinase kinase kinase activity | 56 | 28 | 1.70E-28 |
| MF | GO:0004523 | RNA-DNA hybrid ribonuclease activity | 47 | 26 | 4.40E-28 |
| MF | GO:0004708 | MAP kinase activity | 43 | 23 | 1.60E-24 |
| MF | GO:0004518 | nuclease activity | 448 | 158 | 2.30E-22 |
| MF | GO:0097602 | cullin family protein binding | 21 | 13 | 1.60E-15 |
| MF | GO:0019901 | protein kinase binding | 117 | 25 | 3.90E-15 |
| MF | GO:0052625 | 4-aminobenzoate amino acid synthetase activity | 10 | 8 | 2.10E-11 |
| MF | GO:0052626 | benzoate amino acid synthetase activity | 10 | 8 | 2.10E-11 |
| MF | GO:0052627 | vanillate amino acid synthetase activity | 10 | 8 | 2.10E-11 |
| MF | GO:0052628 | 4-hydroxybenzoate amino acid synthetase activity | 10 | 8 | 2.10E-11 |
| MF | GO:0008146 | sulfotransferase activity | 23 | 10 | 3.30E-10 |
| MF | GO:0080150 | S-adenosyl-L-methionine:benzoic acid carboxyl methyl transferase activity | 13 | 8 | 5.70E-10 |
| MF | GO:0052624 | 2-phytyl-1,4-naphthoquinone methyltransferase activity | 15 | 8 | 2.70E-09 |
| MF | GO:0046872 | metal ion binding | 3823 | 203 | 2.10E-08 |
| MF | GO:0009055 | electron transfer activity | 132 | 15 | 7.20E-06 |
| MF | GO:0003968 | RNA-directed 5'-3' RNA polymerase activity | 39 | 6 | 0.00087 |
| MF | GO:0046933 | proton-transporting ATP synthase activity, rotational mechanism | 27 | 5 | 0.00099 |
| MF | GO:0050135 | NAD(P)+ nucleosidase activity | 106 | 9 | 0.00386 |
| MF | GO:0061809 | NAD+ nucleotidase, cyclic ADP-ribose generating | 106 | 9 | 0.00386 |
| MF | GO:0003730 | mRNA 3'-UTR binding | 21 | 3 | 0.02231 |
| MF | GO:0035064 | methylated histone binding | 26 | 3 | 0.03919 |
| MF | GO:0008143 | poly(A) binding | 26 | 3 | 0.03919 |
| MF | GO:0043531 | ADP binding | 156 | 9 | 0.03977 |
| MF | GO:0003712 | transcription coregulator activity | 136 | 8 | 0.04626 |

Supplementary Table 17 Function description of genes under positive selection in Swiss-prot database

| Gene.ID | Gene name | Function |
| --- | --- | --- |
| Esa00505.t1 | VIP3 | Component of the PAF1 complex (PAF1C) which is involved in histone modifications such as methylation on histone H3 'Lys-4' (H3K4me3) (PubMed:20363855). Involved in regulation of flowering time. Required for the expression of the flowering repressor and MADS box gene FLC (PubMed:12750345, PubMed:18725930). Required for histone H3 trimethylation on 'Lys-4' (H3K4me3) and histone demethylation on 'Lys-36' (H3K36me2) at the FLC locus. Prevents trimethylation on 'Lys-27' (H3K27me3) at the same locus (PubMed:18725930). Not required for meiotic recombination or progression (PubMed:16716192).Component of the SKI complex which is thought to be involved in exosome-mediated RNA decay and associates with transcriptionally active genes in a manner dependent on PAF1 complex (PAF1C) (PubMed:22511887).Required for proper progression of cell differentiation process (PubMed:23134555). |
| Esa00554.t1 | PUB3 | Functions as an E3 ubiquitin ligase |
| Esa02335.t1 | EC:1.1.1.27 | Catalytic Activity |
| Esa08830.t1 | At3g16010 | Showing features for repeat |
| Esa11267.t1 | TOR1L5 | microtubule binding |
| Esa17404.t1 | NA | NA |
| Esa18399.t1 | At1g66310 | Showing features for domain |
| Esa19539.t3 | NA | NA |
| Esa20545.t1 | NA | NA |
| Esa20905.t1 | ETFA | The electron transfer flavoprotein serves as a specific electron acceptor for several dehydrogenases, including five acyl-CoA dehydrogenases, glutaryl-CoA and sarcosine dehydrogenase. It transfers the electrons to the main mitochondrial respiratory chain via ETF-ubiquinone oxidoreductase (ETF dehydrogenase) (By similarity). |
| Esa21072.t1 | At4g01400 | Group II intron splicing; mitochondrial respiratory chain complex I assembly; mitochondrial RNA processing |
| Esa21515.t1 | At5g05200 | ATP binding; kinase activity; lipid homeostasis; mitochondrion organization |
| Esa23191.t1 | Os09g0364000 | Showing features for region, zinc finger. |

Supplementary Table 18 GO enrichment results of positive selection genes in *E. salsugineum*

| Class | GO.ID | Term | Annotated | Significant | Classic  Fisher |
| --- | --- | --- | --- | --- | --- |
| BP | GO:0009826 | unidimensional cell growth | 360 | 3 | 0.004 |
| BP | GO:0052324 | plant-type cell wall cellulose biosynthetic process | 15 | 1 | 0.0071 |
| BP | GO:0006108 | malate metabolic process | 15 | 1 | 0.0071 |
| BP | GO:2001006 | regulation of cellulose biosynthetic process | 16 | 1 | 0.0076 |
| BP | GO:0007019 | microtubule depolymerization | 17 | 1 | 0.008 |
| BP | GO:0051013 | microtubule severing | 18 | 1 | 0.0085 |
| BP | GO:1990778 | protein localization to cell periphery | 21 | 1 | 0.0099 |
| BP | GO:0006744 | ubiquinone biosynthetic process | 21 | 1 | 0.0099 |
| BP | GO:0015996 | chlorophyll catabolic process | 25 | 1 | 0.0118 |
| BP | GO:0033365 | protein localization to organelle | 293 | 2 | 0.0152 |
| BP | GO:0045037 | protein import into chloroplast stroma | 35 | 1 | 0.0165 |
| BP | GO:0008360 | regulation of cell shape | 35 | 1 | 0.0165 |
| BP | GO:0006635 | fatty acid beta-oxidation | 37 | 1 | 0.0174 |
| BP | GO:0006099 | tricarboxylic acid cycle | 50 | 1 | 0.0235 |
| BP | GO:0007275 | multicellular organism development | 3179 | 3 | 0.0392 |
| BP | GO:0010224 | response to UV-B | 91 | 1 | 0.0423 |
| CC | GO:0010005 | cortical microtubule, transverse to long axis | 14 | 1 | 0.0069 |
| CC | GO:0008352 | katanin complex | 15 | 1 | 0.0074 |
| CC | GO:0055028 | cortical microtubule | 32 | 2 | 0.0078 |
| CC | GO:1904949 | ATPase complex | 28 | 1 | 0.0137 |
| CC | GO:0010319 | stromule | 36 | 1 | 0.0176 |
| CC | GO:0009706 | chloroplast inner membrane | 94 | 1 | 0.0454 |
| CC | GO:0080008 | Cul4-RING E3 ubiquitin ligase complex | 97 | 1 | 0.0469 |
| MF | GO:0008017 | microtubule binding | 178 | 3 | 0.00057 |
| MF | GO:0016464 | chloroplast protein-transporting ATPase activity | 11 | 1 | 0.0052 |
| MF | GO:0016615 | malate dehydrogenase activity | 14 | 1 | 0.0066 |

Supplementary Table 19 The result of annotations of positive selection genes in *E. salsugineum* via blast research

| Query id | Subject id | pident | length | Mis  match | gap open | q.start | q.end | s.start | s.end | e-value | bit  score |
| --- | --- | --- | --- | --- | --- | --- | --- | --- | --- | --- | --- |
| Esa00505.t1 | Q8H0T9 | 32.70 | 159 | 97 | 2 | 272 | 430 | 60 | 208 | 6.5e-13 | 71.2 |
| Esa00554.t1 | F4I718 | 92.41 | 2135 | 161 | 1 | 3 | 2136 | 1 | 2135 | 0 | 3.98e+03 |
| Esa02335.t1 | Q9SN86 | 89.43 | 388 | 37 | 2 | 3 | 386 | 4 | 391 | 0 | 678 |
| Esa08830.t1 | O82178 | 90.68 | 590 | 52 | 1 | 12 | 598 | 1 | 590 | 0 | 1.12e+03 |
| Esa11267.t1 | Q9T041 | 89.09 | 880 | 80 | 1 | 1 | 880 | 1 | 864 | 0 | 1.6e+03 |
| Esa18399.t1 | Q9FZ70 | 90.39 | 416 | 40 | 0 | 15 | 430 | 1 | 416 | 0 | 764 |
| Esa20905.t1 | Q9C6I6 | 91.74 | 363 | 28 | 1 | 1 | 361 | 1 | 363 | 0 | 669 |
| Esa21072.t1 | Q9SSR6 | 91.14 | 519 | 45 | 1 | 1 | 518 | 1 | 519 | 0 | 966 |
| Esa21515.t1 | Q9SBB2 | 87.15 | 638 | 63 | 7 | 1 | 634 | 1 | 623 | 0 | 1.11e+03 |
| Esa23191.t1 | Q84WU9 | 91.44 | 596 | 50 | 1 | 1 | 595 | 1 | 596 | 0 | 1.13e+03 |

**Supplementary Table 20 22 blocks in the genome of *Arabidopsis thaliana***

| AK | AT | Genomic blocks | Start gene | End gene | BAC clone |
| --- | --- | --- | --- | --- | --- |
| AK1 | AT1 | A | AT1G01010 | AT1G19840 | F14P1 |
|  | AT1 | B | AT1G19850 | AT1G37130 | F12K21 |
|  | AT1 | C | AT1G43020 | AT1G56190 | T6H22 |
| AK2 | AT1 | D | AT1G64670 | AT1G56210 | F14G9 |
|  | AT1 | E | AT1G64960 | AT1G80950 | F23A5 |
| AK3 | AT3 | F | AT3G01015 | AT3G25520 | MWL2 |
|  | AT2 | G | AT2G05170 | AT2G07690 | T25N22 |
|  | AT2 | H | AT2G10940 | AT2G20900 | F5H14 |
| AK4 | AT2 | I | AT2G20920 | AT2G31035 | T19L18 |
|  | AT2 | J | AT2G31040 | AT2G48150 | T8I13 |
| AK5 | AT2 | K-L | AT2G01060 | AT2G05160 | F3C11 |
|  | AT3 | K-L | AT3G25540 | AT3G35960 | T4A2 |
|  | AT3 | M-N | AT3G42180 | AT3G63530 | F16M2 |
| AK6 | AT4 | O | AT4G00026 | AT4G05450 | T1J1 |
|  | AT4 | P | AT4G12620 | AT4G07390 | T3H13 |
|  | AT5 | Q | AT5G30510 | AT5G23010 | T20O7 |
|  | AT5 | R | AT5G23000 | AT5G01010 | F7J8 |
| AK7 | AT5 | S | AT5G42110 | AT5G32470 | F5H8 |
|  | AT4 | T | AT4G12700 | AT4G16240 | F18A5 |
|  | AT4 | U | AT4G16250 | AT4G40100 | T5J17 |
| AK8 | AT5 | V | AT5G47810 | AT5G42130 | MJC20 |
|  | AT5 | W | AT5G47820 | AT5G60800 | MUP24 |
|  | AT5 | X | AT5G60805 | AT5G67640 | K9I9 |


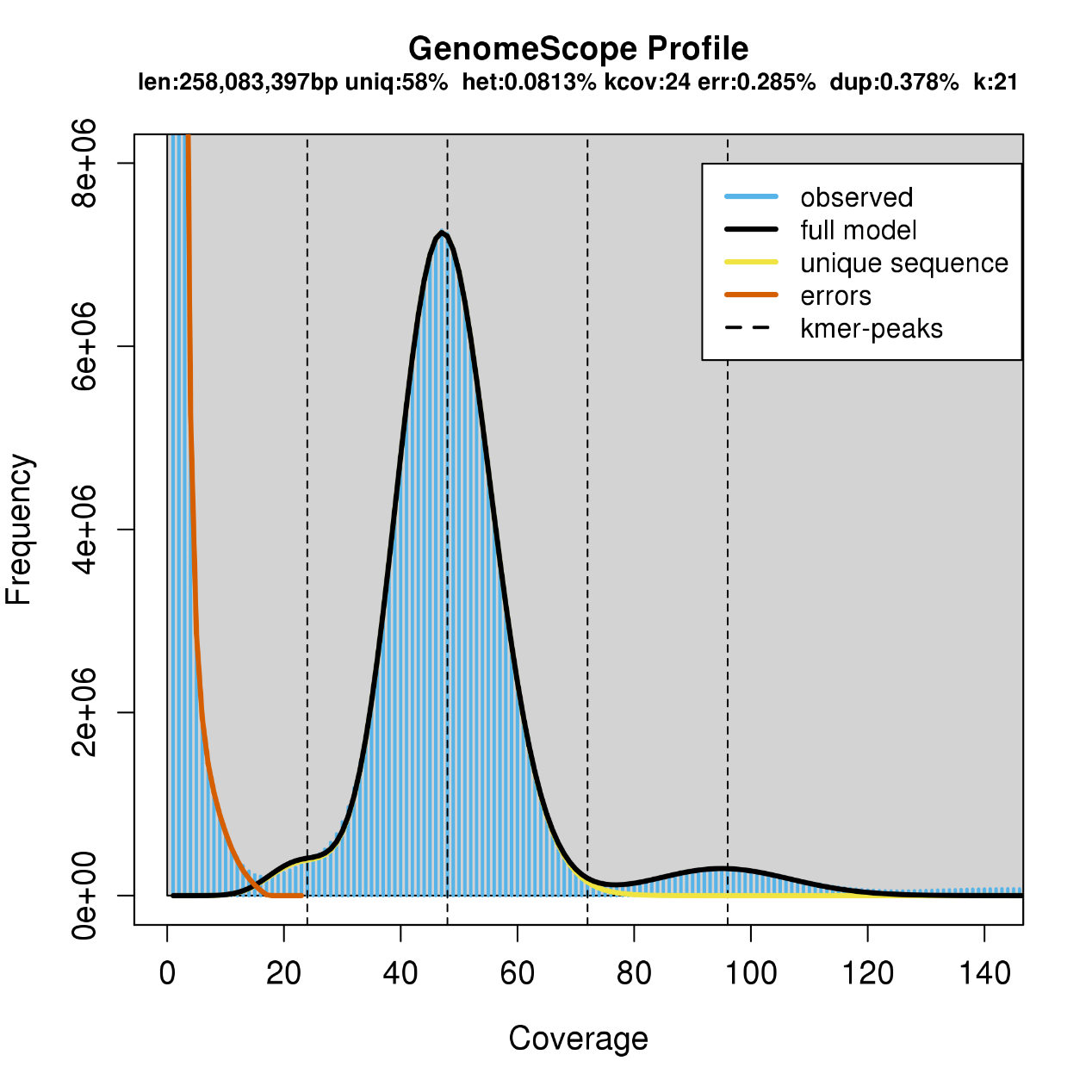


Supplement Fig. 1 The K-mer analysis used to estimate *E. salsugineum* genome size


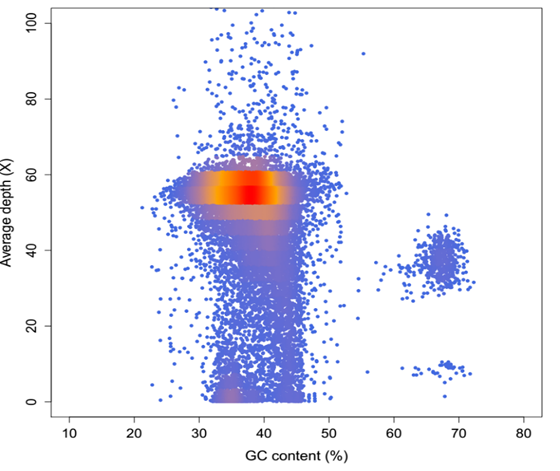


Supplement Fig. 2 GC content and sequencing depth distribution


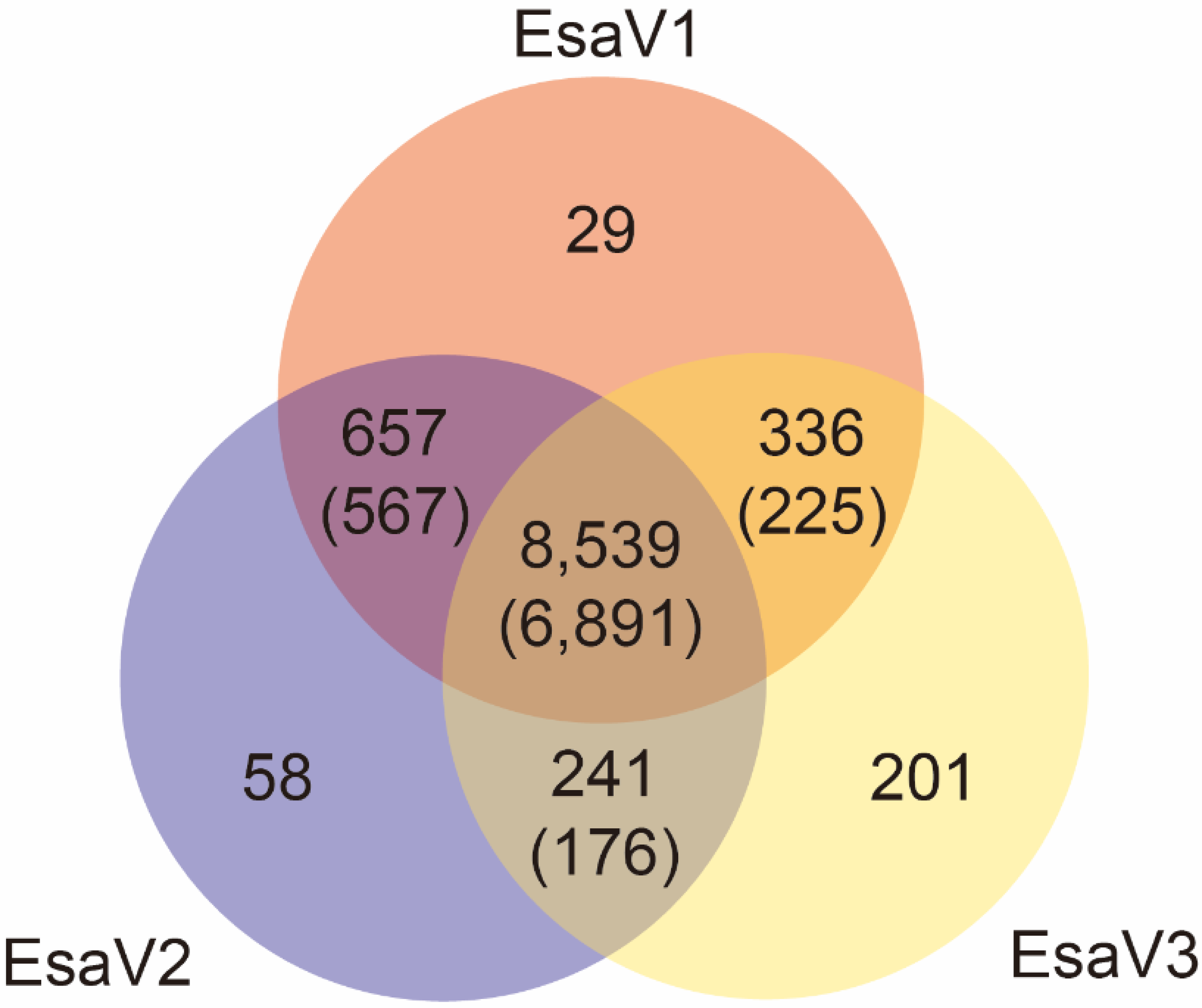


**Supplement Fig. 3 Gene content difference between three versions of *E. salsugineum* genome. Numbers of gene families were shown, and numbers in parentheses indicate gene families with conserved copy number between different versions.**

**
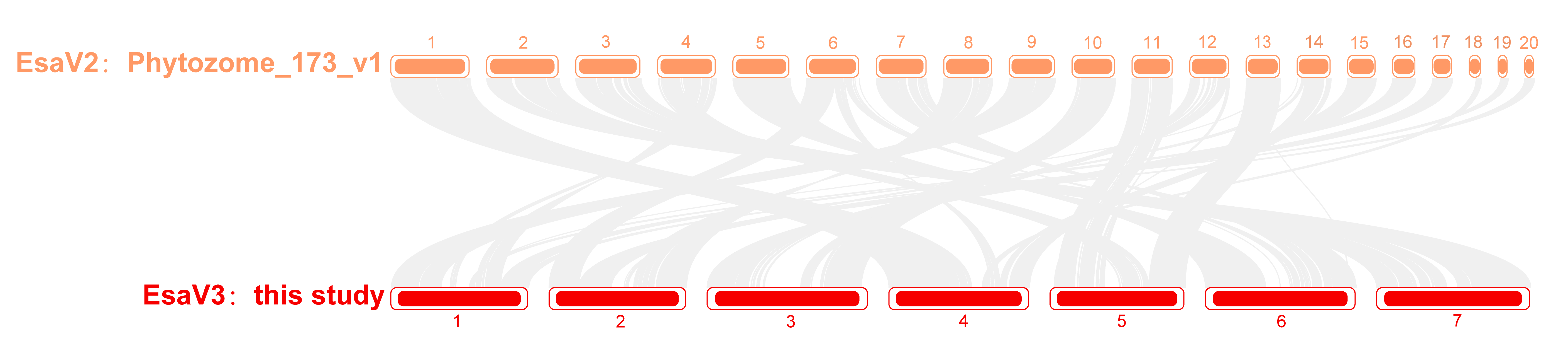
**

**Supplement Fig. 4** **Synteny plot of gene pairs on whole-genome wide for EsaV2 and EsaV3. EsaV2 genome was not assembled at chromosome-level, so Number 1 actually stands for scaffold_1, Number 2 actually stands for scaffold_2 and so on in EsaV2.**

**
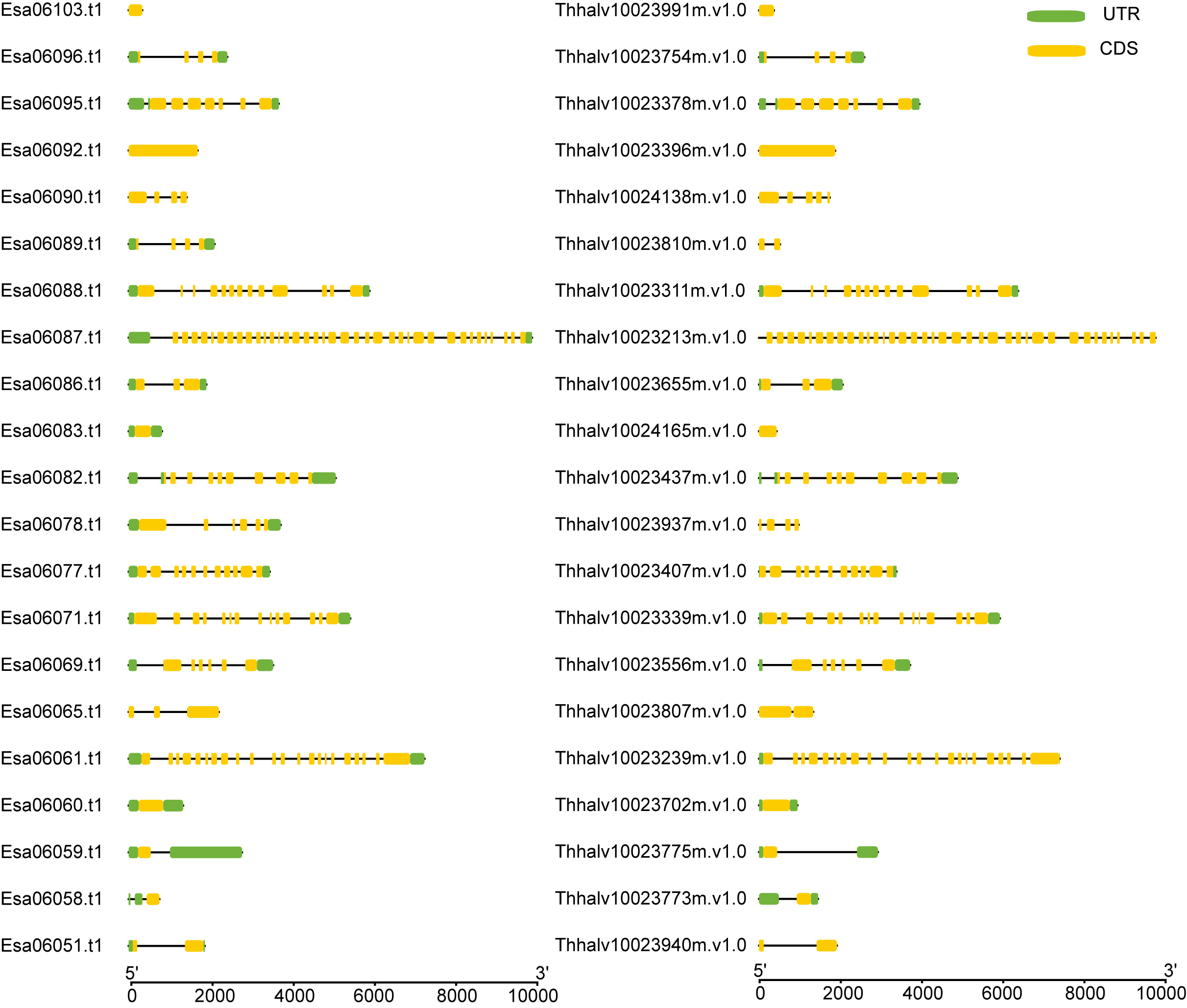
**

**Supplement Fig. 5** **Gene structure of synteny gene pairs of EsaV2 and EsaV3 (part).**

**
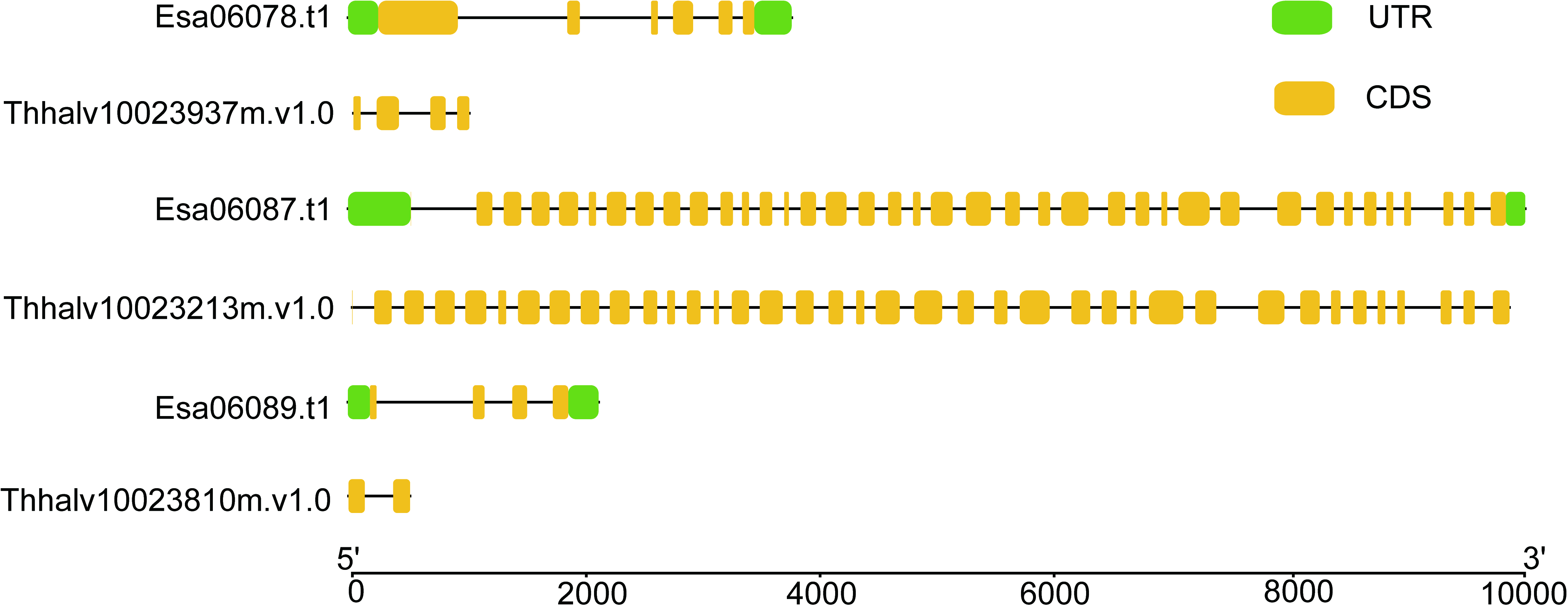
**

**Supplement Fig. 6 Obvious gene structure comparison of EsaV2 and EsaV3 (part).**

**
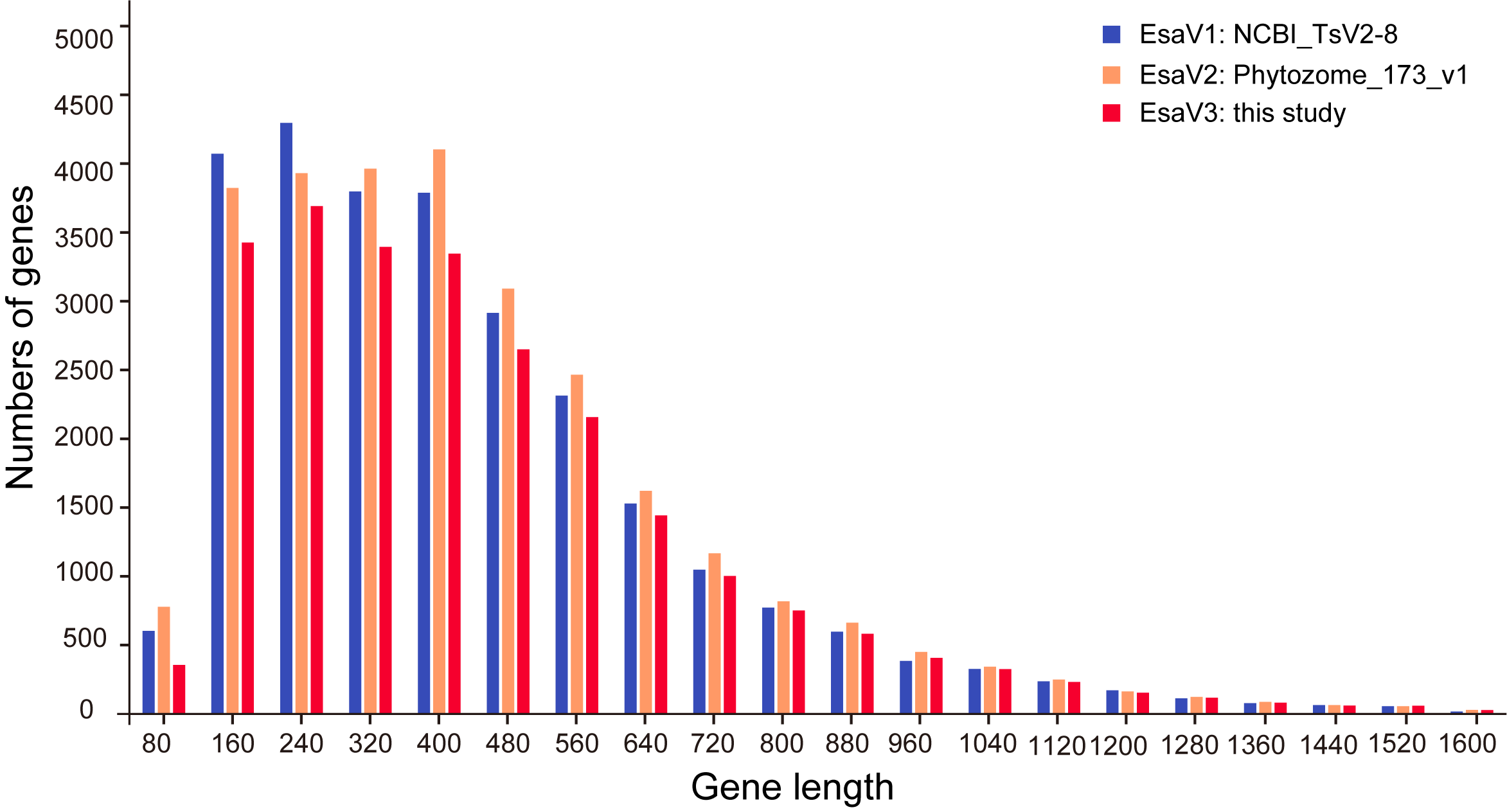
**

**Supplement Fig. 7** **Gene length statistics for three *E. salsugineum* assemblies**

**
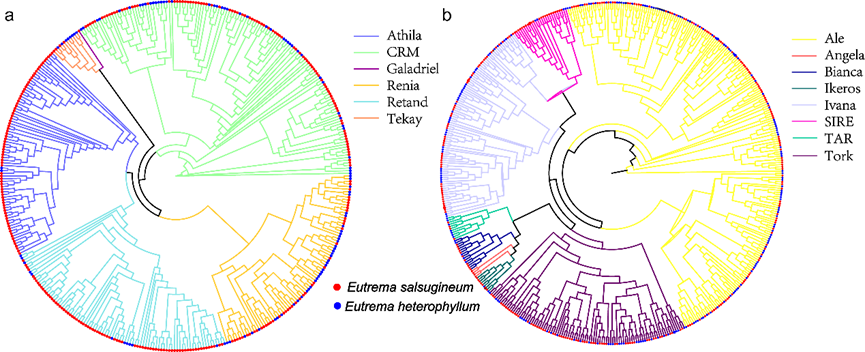
**

**Supplemental Fig 8** Phylogenetic trees of LTR/Gypsy (a) and LTR/Copia (b) based on the reverse transcriptase domains of LTR-RTs with *E. salsugineum* and *E. heterophyllum* together. The red and blue circle represent the sequence from *E. salsugineum* and *E. heterophyllum,* respectively.

**
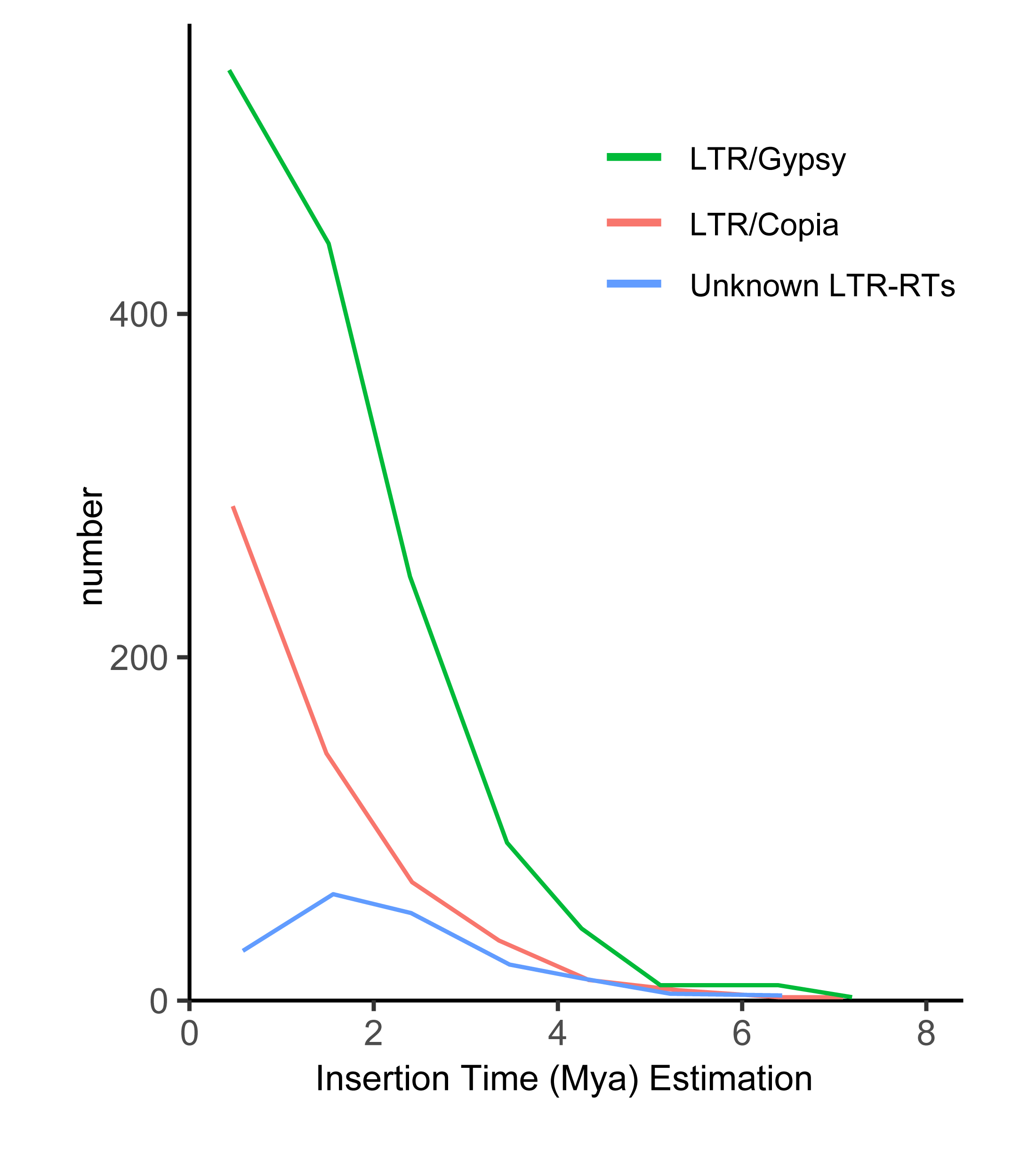
**

**Supplement Fig. 9 Estimated insertion times of LTR/Gypsy, LTR/Copia, and an unknown LTR retrotransposons in** ***E. salsugineum*.**

**

**

**Supplement Fig. 10 Gene Ontology enrichment of genes near the location of recent LTR insertion.**

**

**

**Supplement Fig. 11** **Gene density map of 1,153 newly annotated genes in *E. salsugineum*** **distributed on seven chromosomes.**

**

**

**Supplement Fig. 12 Gene structure of 1,153 newly annotated genes in *E. salsugineum* (part).**

**
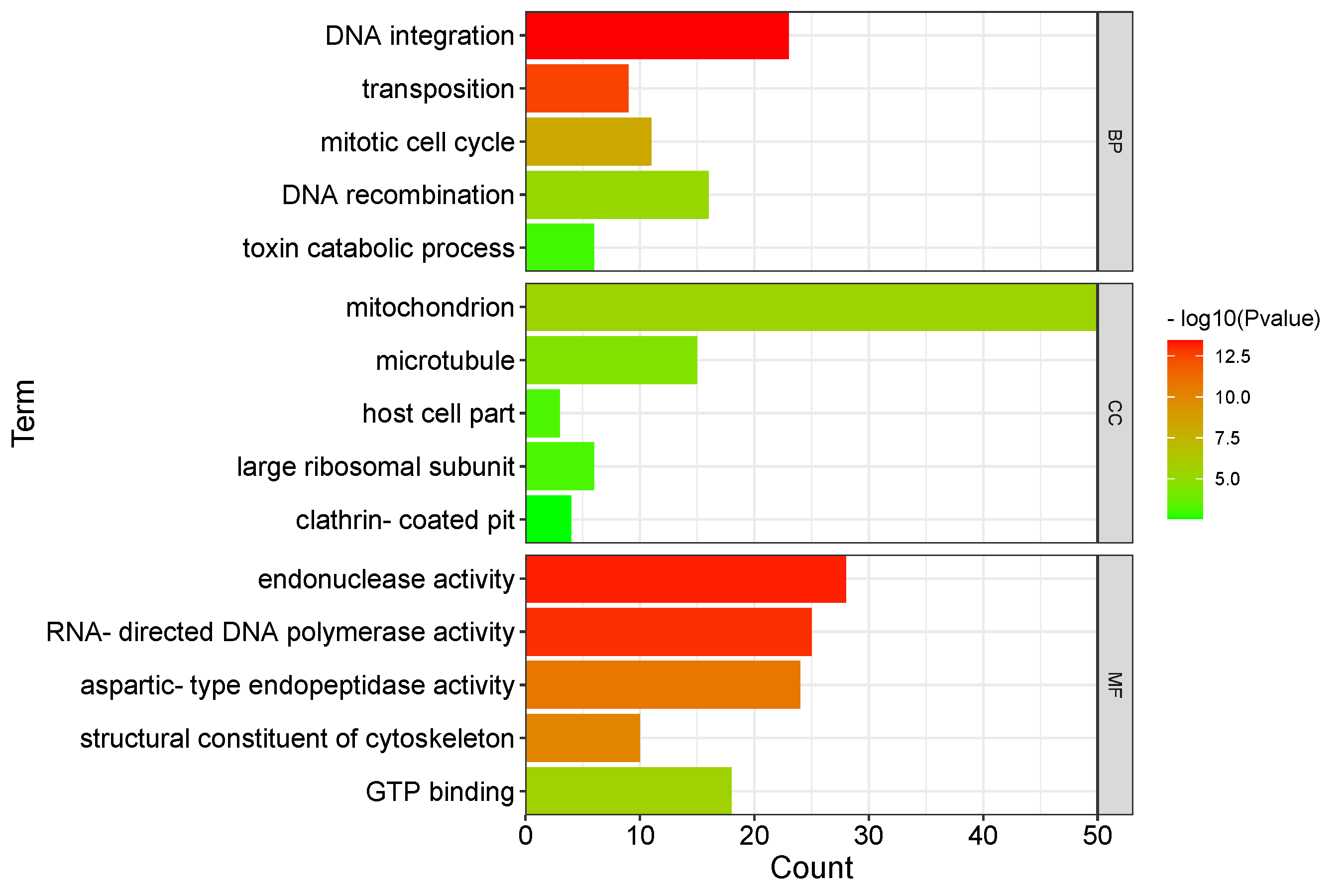
**

**Supplement Fig. 13 Gene Ontology enrichment of 1,153 newly annotated genes in *E. salsugineum*.**

**
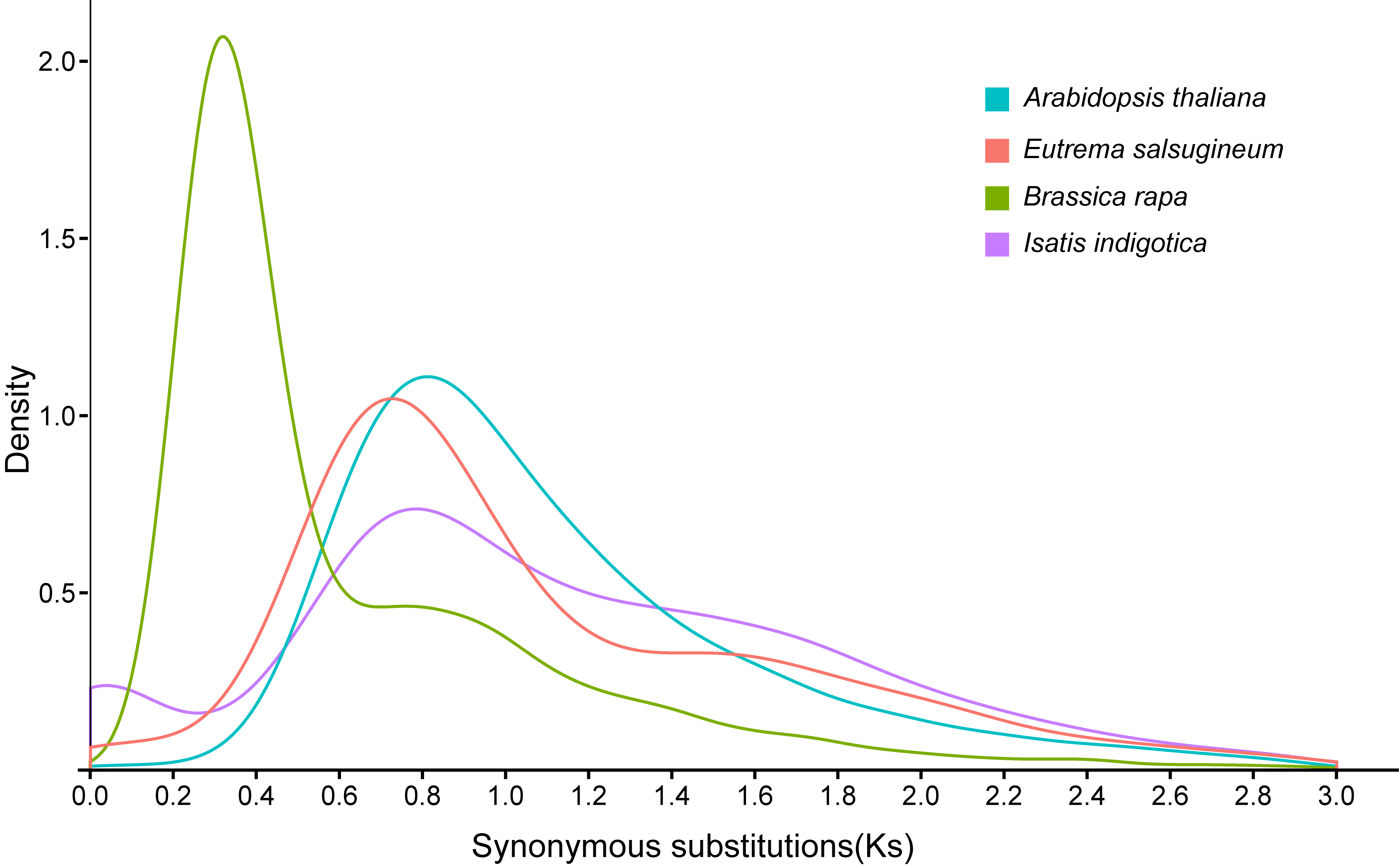
**

**Supplement Fig. 14 Distribution of the synonymous substitution rate (Ks) between *A. thaliana*, *E. salsugineum*, *B. rapa*, and *I. indigotica*.**

**
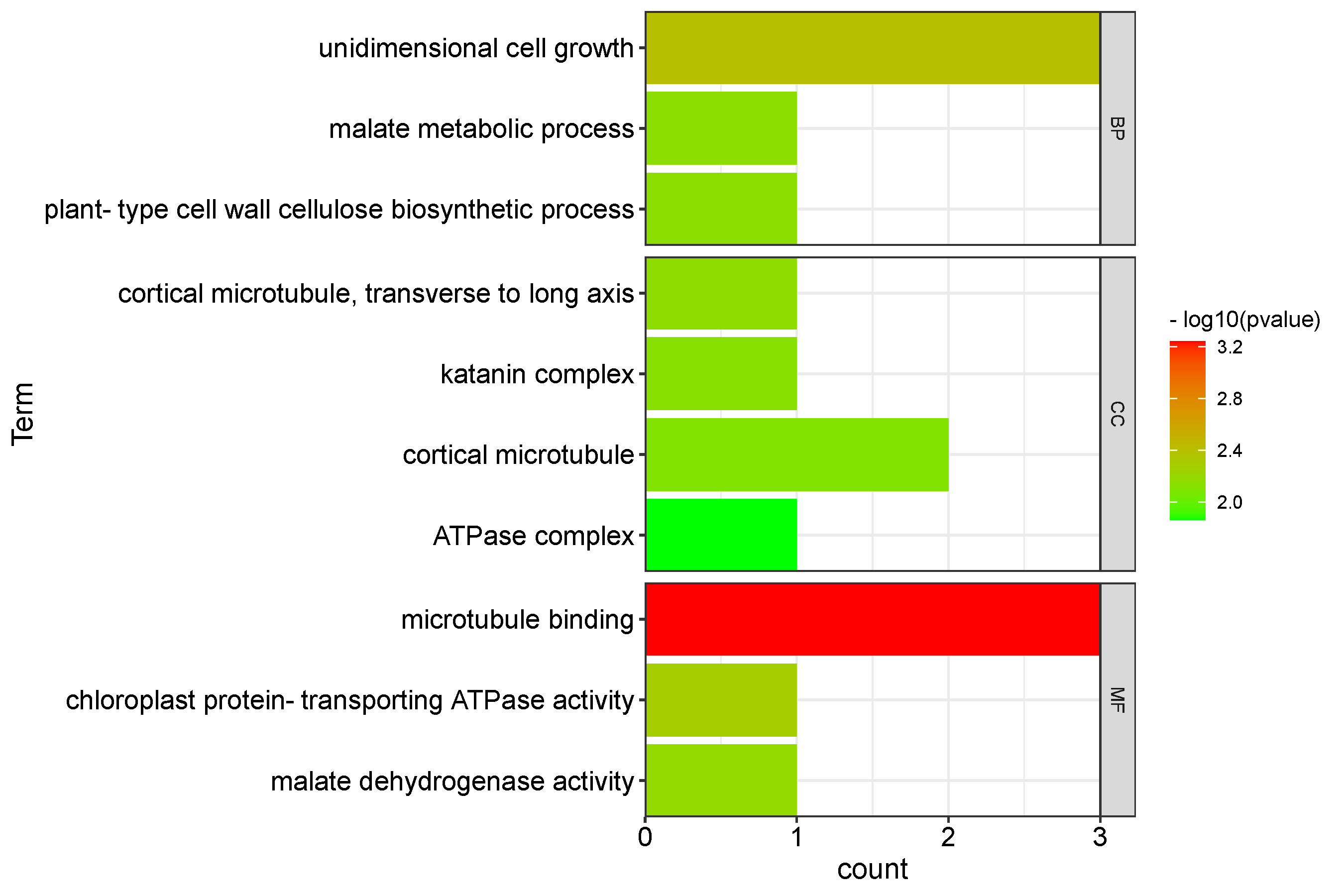
**

**Supplement Fig. 15 Gene Ontology enrichment of positively selected genes in *E. salsugineum*.**

**
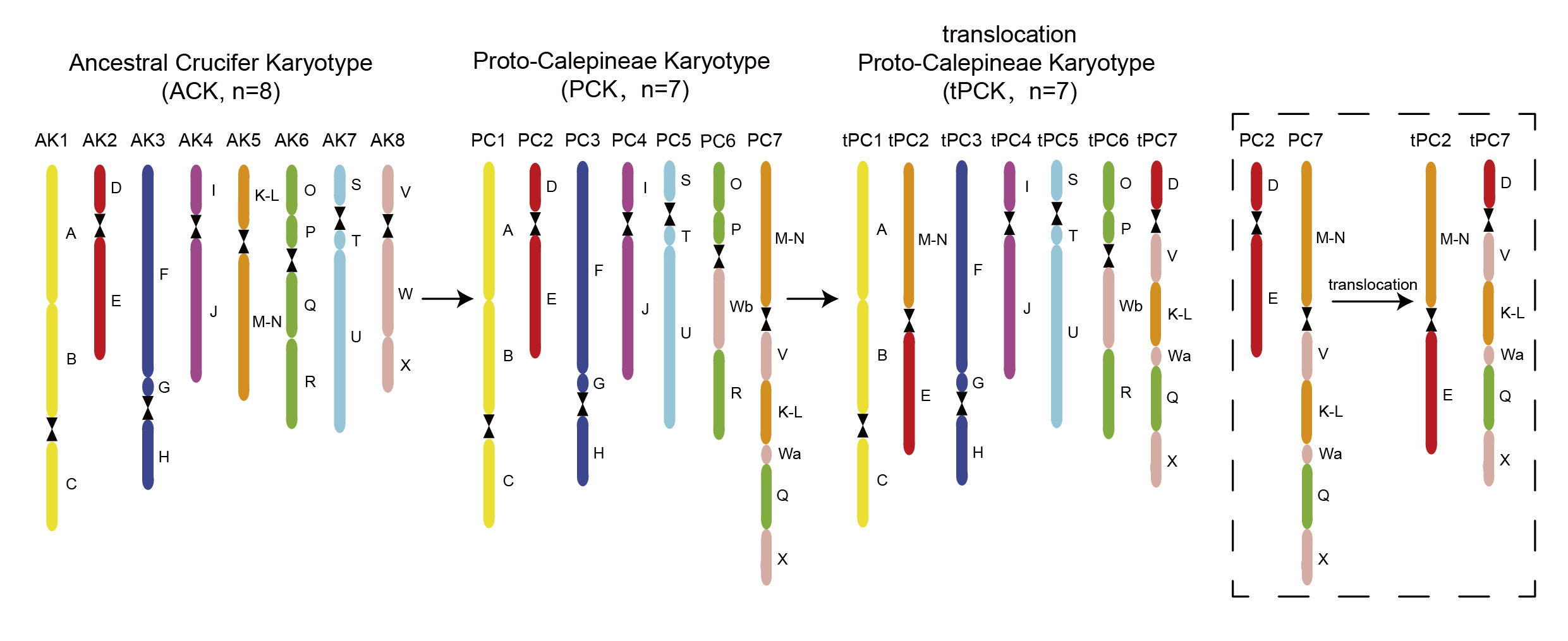
**

**Supplement Fig. 16 The evolution of tPCK karyotype of *E. salsugineum*.**


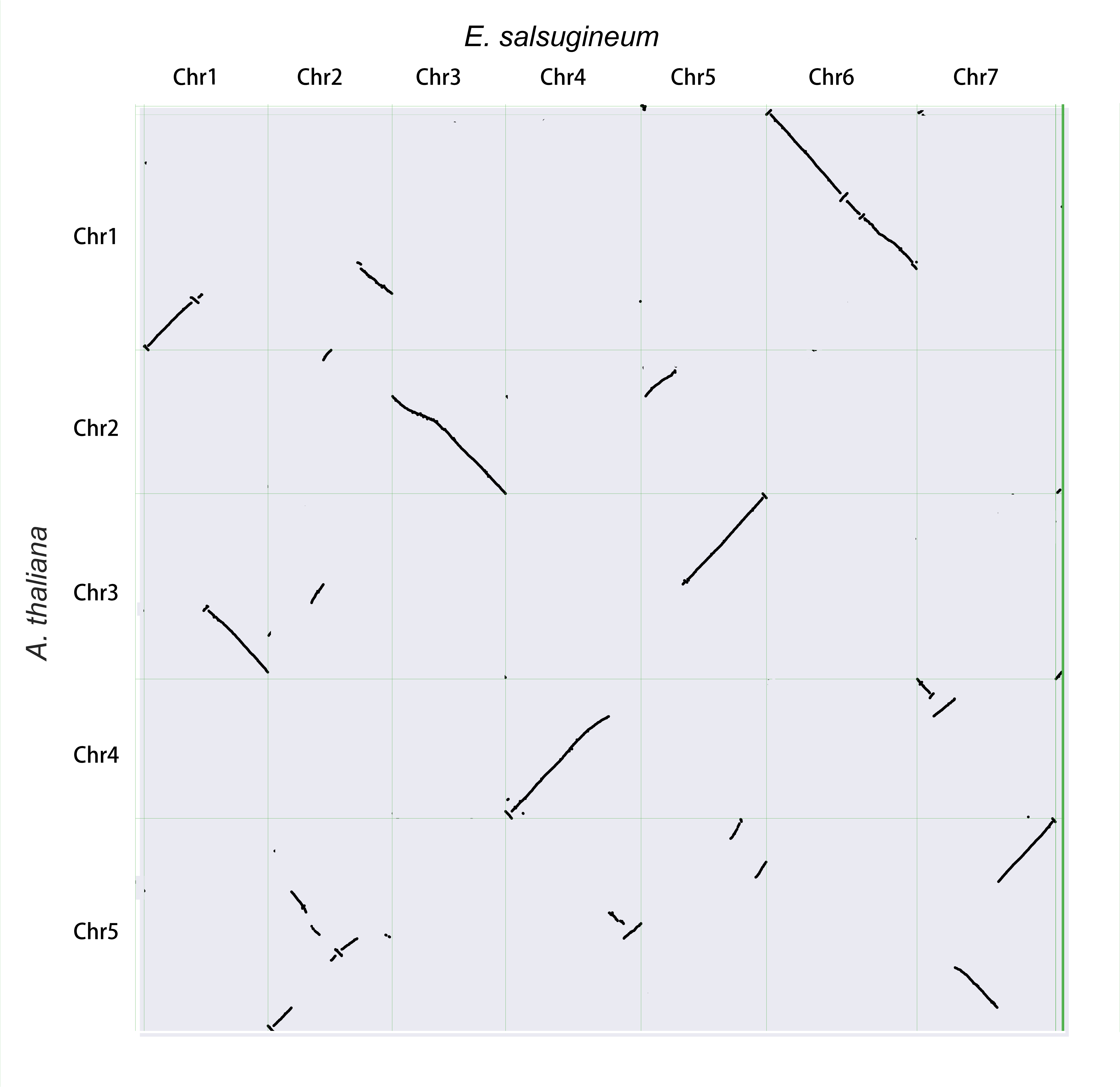


**Supplement Fig. 17 The dot plot between *E. salsugineum* and *A. thaliana*.**
